# Defect‐Engineered Microwave‐Responsive Ni@C Composites From Waste PET for Catalytic Plastic Upcycling

**DOI:** 10.1002/adma.74578

**Published:** 2026-08-12

**Authors:** Xiao Lu, Shiying Ren, Jingkai Lin, Kunsheng Hu, Yalan Mo, Shaobin Wang, Wenjie Tian, Xiaoguang Duan

**Affiliations:** ^1^ School of Chemical Engineering Adelaide University Adelaide South Australia Australia

**Keywords:** catalysis, defects, microwave, plastics, upcycling

## Abstract

Direct catalytic upcycling of solid plastic waste is challenging owing to its chemical robustness, and existing conversion routes often require harsh conditions or costly catalysts. Here, we develop a circular plastic‐to‐catalyst‐to‐product strategy converting waste poly(ethylene terephthalate) (PET) bottles into a microwave‐responsive composite catalyst for microwave‐assisted catalytic plastic upcycling. First, microwave‐assisted PET depolymerization and Ni‐MOF nanorod crystallization generate abundant missing‐cluster defects, inherited during pyrolysis as lattice‐distorted Ni nano‐cores and edge dislocations encapsulated within a defective carbon shell (Ni@C). These strain‐rich Ni─C heterointerfaces enhance dielectric loss and interfacial polarization under microwave irradiation, promoting local microwave energy dissipation at catalyst‐plastic contacts and accelerating peroxymonosulfate (PMS) activation. Coupled microwave‐thermal‐chemical PMS activation initiates polymer‐chain disordering, hydrogen abstraction, and C─C bond scission in high‐density polyethylene (HDPE) particles before oxidative functionalization, making it more efficient than oxidation‐dominated hydrothermal heating. Spectroscopic and strain‐mapping analyses reveal that dislocation‐rich Ni cores and carbon defects govern microwave energy dissipation and thus catalytic oxidation reactivity. The optimized Ni@C catalyst achieves up to 96% degradation of HDPE and converts products into valuable liquid hydrocarbons and oxygenates with limited phytotoxicity. Overall, this work integrates waste‐derived catalyst design with microwave‐assisted plastic conversion, offering a route toward circular plastic upcycling and carbon recovery.

## Graphical abstract

A circular upcycling strategy transforms waste poly(ethylene terephthalate) bottles into microwave‐responsive Ni@C catalyst featuring lattice‐distorted nickel cores and defective carbon shells. These strain‐rich heterointerfaces enhance dielectric loss and promote localized microwave hotspots, accelerating peroxymonosulfate activation and polymer‐chain activation during high‐density polyethylene conversion into valuable hydrocarbons and oxygenates. This work offers a sustainable route for plastic‐to‐catalyst design and carbon recovery.

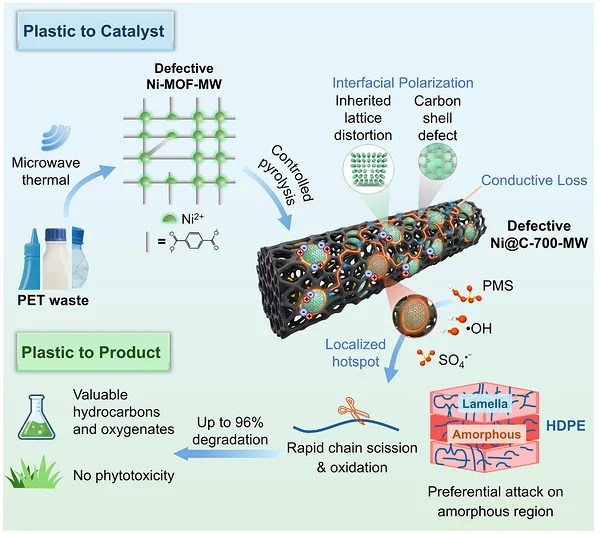

## 1 Introduction

Global plastic production reached 430.9 million tons in 2024, yet only about 14% is effectively recycled, leading to the pervasive accumulation of microplastics (MPs) in aquatic and terrestrial environments [1, 2]. These MPs, defined as having a size smaller than 5 mm, can accumulate in food chains, concentrate toxic pollutants, and impose physical and chemical hazards to organisms [3]. Polyolefins such as high‐density polyethylene (HDPE), low‐density polyethylene (LDPE), and polypropylene (PP) account for two‐thirds of global plastic production and dominate environmental MPs because of their extensive use, chemical inertness, and resistance to natural degradation [4]. Conventional plastic waste disposal methods, such as landfilling and incineration, face significant limitations [5, 6]. Landfilling leads to long‐term environmental accumulation and potential soil and groundwater contamination, while incineration involves high energy consumption and the risk of toxic emissions. Catalytic upcycling of plastic waste provides a source‐control route of MPs by converting polymer feedstocks into useful products [7]. Current chemical upcycling routes for bulk plastics, including pyrolysis, hydrogenolysis, and tandem alkane metathesis, typically require high temperatures, elevated pressures, or precious metal catalysts [8, 9, 10], limiting their applicability for plastic valorization. Thus, energy‐efficient and selective plastic transformation approaches capable of operating under mild conditions remain a critical challenge.

Microwave (MW) irradiation offers volumetric heating and the generation of localized hotspots via dipolar polarization, enabling rapid bond cleavage of hydrocarbons with high energy utilization efficiency [9, 11]. However, most microwave‐assisted processes still rely on high bulk reaction temperatures, typically exceeding 280°C [3, 9], while the structure‐function relationships governing microwave energy conversion at catalytic interfaces remain poorly understood [12]. Meanwhile, Fenton and Fenton‐like systems utilize reactive oxygen species (ROS) to oxidatively degrade polymers under relatively mild hydrothermal conditions (120‐180°C) [13, 14, 15, 16, 17]. Combining microwave energy input with Fenton‐like oxidation may provide a complementary approach to enable synergistic thermal‐chemical pathways for plastic transformations. Nevertheless, catalyst architectures capable of coupling microwave energy dissipation with efficient peroxide activation have not been rationally designed.

Addressing this gap motivates the rational design of catalysts capable of integrating microwave energy conversion with catalytic activity in Fenton‐like reactions. Metal‐core @ carbon‐shell (M@C) structures provide a promising platform to deliver such synergy. Metal‐carbon heterointerfaces with enhanced microwave absorption and magnetic‐dielectric coupling promote dielectric polarization and electromagnetic energy attenuation, thereby facilitating efficient energy dissipation and localized hot‐spot generation [18, 19]. Incorporation of structural and surface defects into such hybrid structures further boosts microwave responsiveness, energy attenuation, and catalytic activity in Fenton‐like chemistry [18, 20, 21]. In this regard, metal organic frameworks (MOFs) are ideal precursors for microwave‐responsive metal@carbon composite catalysts, as their uniform metal–ligand coordination yields highly dispersed metal species [22, 23, 24]. However, the high cost of traditional MOF synthesis remains a barrier for large‐scale deployment. Poly(ethylene terephthalate) (PET), an abundant industrial waste stream, has emerged as a sustainable precursor for MOF fabrication, opening the possibility of plastic‐to‐catalyst conversions [25, 26]. PET‐derived MOFs have demonstrated promising applications in energy conversion and storage [27], photocatalysis [28] and pollutant degradation [25]. However, a closed‐loop plastic‐to‐catalyst system for plastics conversion that unifies defect engineering, microwave energy transduction, and catalytic advanced oxidation has not been demonstrated.

Building on this concept, we developed a closed‐loop strategy by upcycling waste PET into a Ni‐based MOF (Ni‐MOF) via microwave‐assisted synthesis, which was subsequently converted into defect‐rich Ni@C composites via controlled pyrolysis. Microwave processing induces missing‐cluster defects in Ni‐MOFs which evolve into lattice‐distorted Ni cores and defective graphitic shells, forming abundant strain‐rich metal‐carbon interfaces. Such features maximize dielectric loss and promote interfacial polarization under microwave irradiation, thereby accelerating peroxymonosulfate (PMS) activation and yielding diverse reactive oxygen species (ROS). The resulting dual pathway, including localized hotspot‐induced plastic decrystallization and C─C bond scission coupled with ROS oxidation, enables up to 96% degradation of HDPE with simultaneous formation of value‐added products. This work demonstrates a closed‐loop pathway that integrates plastic upcycling into a microwave‐responsive catalyst for microplastic degradation via microwave‐assisted catalytic oxidation and, more importantly, highlights how plastic waste can be used for circular microplastic remediation.

## 2 Results and Discussion

### 2.1 Defect Evolution in Microwave‐Derived Ni‐MOF and Ni@C Catalysts

The microwave‐responsive catalyst was constructed through a two‐stage defect‐engineering strategy (Figure 1). Microwave irradiation volumetrically heats the mixed polar *N*,*N*‐dimethylformamide (DMF)/H_2_O medium (at a volume ratio of 2:1), accelerating PET depolymerization into 1,4‐benzenedicarboxylic acid (H_2_BDC), which is rapidly deprotonated to the BDC anion linker; this process simultaneously drives rapid Ni‐MOF nucleation [25]. The resulting metal‐rich but linker‐limited reaction environment created under microwave conditions lowers the nucleation energy barrier and promotes the rapid formation of both fully coordinated and undercoordinated metal nodes [29, 30, 31]. The missing‐cluster defects are irreversibly incorporated within the Ni‐MOF‐MW structure during crystallization [32].

**FIGURE 1:**
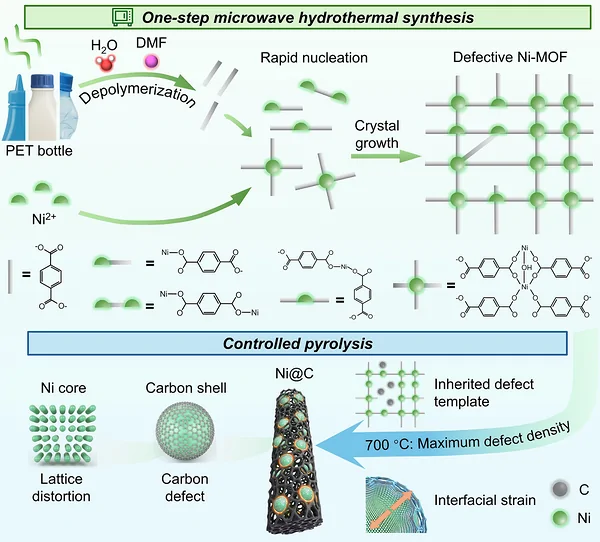
Schematic illustration of two‐stage defect engineering route from waste PET to a microwave‐responsive Ni@C‐MW catalyst: microwave‐assisted PET depolymerization and Ni‐MOF‐MW formation under a metal‐rich, linker‐limited environment (upper); controlled pyrolysis inducing the transformation of defective Ni‐MOF‐MW into a defect‐rich Ni@C‐MW structure (lower).

During controlled pyrolysis, under‐coordinated linker sites decompose preferentially, creating spatial heterogeneity in the reduction of Ni^2+^ and in the spontaneous carbonization of the organic ligand, forming vacancies [33, 34]. Ni atoms migrate along the MOF‐derived carbon matrix while catalytically graphitizing the carbon shells surrounding the metallic Ni° cores [35]. Meanwhile, the lattice mismatch and thermal contraction between the Ni core and graphitic shell impose interfacial strain, leading to pronounced lattice distortion in Ni^0^ and the generation of edge dislocations [36]. In this way, molecular‐scale defects in the precursor Ni‐MOF‐MW are translated into coupled Ni^0^ lattice and interfacial disorders in the resulting Ni@C‐MW composite, creating a defect‐rich architecture optimized for microwave energy dissipation and thus for microwave‐assisted Fenton‐like catalysis.

To examine the effect of microwave irradiation on defect formation, a hydrothermally synthesized counterpart (Ni‐MOF‐HT) was prepared as a control under otherwise identical conditions. The abbreviations for all materials and reaction systems are summarized in Table S1. X‐ray diffraction (XRD) patterns show that both materials retain the same crystallographic framework, which matches well with the simulated stacking model based on the CCDC 985792 crystal data (Figure S2a) [37]. However, Ni‐MOF‐MW exhibits broader, slightly right‐shifted peaks, implying reduced crystallite size, lattice contraction, and higher microstrain density (Figure S2b).

The two‐dimensional layer morphology of Ni‐MOF‐MW is clearly observed in integrated differential phase contrast‐scanning transmission electron microscopy (iDPC‐STEM) and scanning electron microscopy (SEM) images (Figure 2a,b and Figure S4). Figure 2b shows a cluster‐missing region in Ni‐MOF‐MW, indicating the structural defects induced by local supersaturation and hotspots during microwave synthesis. The SEM image of Ni‐MOF‐MW shows multiple short rods, consistent with rapid nucleation under localized microwave hotspots (Figure S4) [38]. Such nonequilibrium growth conditions are known to promote coordination vacancies and framework disorder in MOF. Consistent with these defects, Ni‐MOF‐MW possesses nearly two‐fold higher surface area, increased pore volume, and a distinct shift toward larger mesopores, compared with Ni‐MOF‐HT (Figure S5 and Table S2).

**FIGURE 2:**
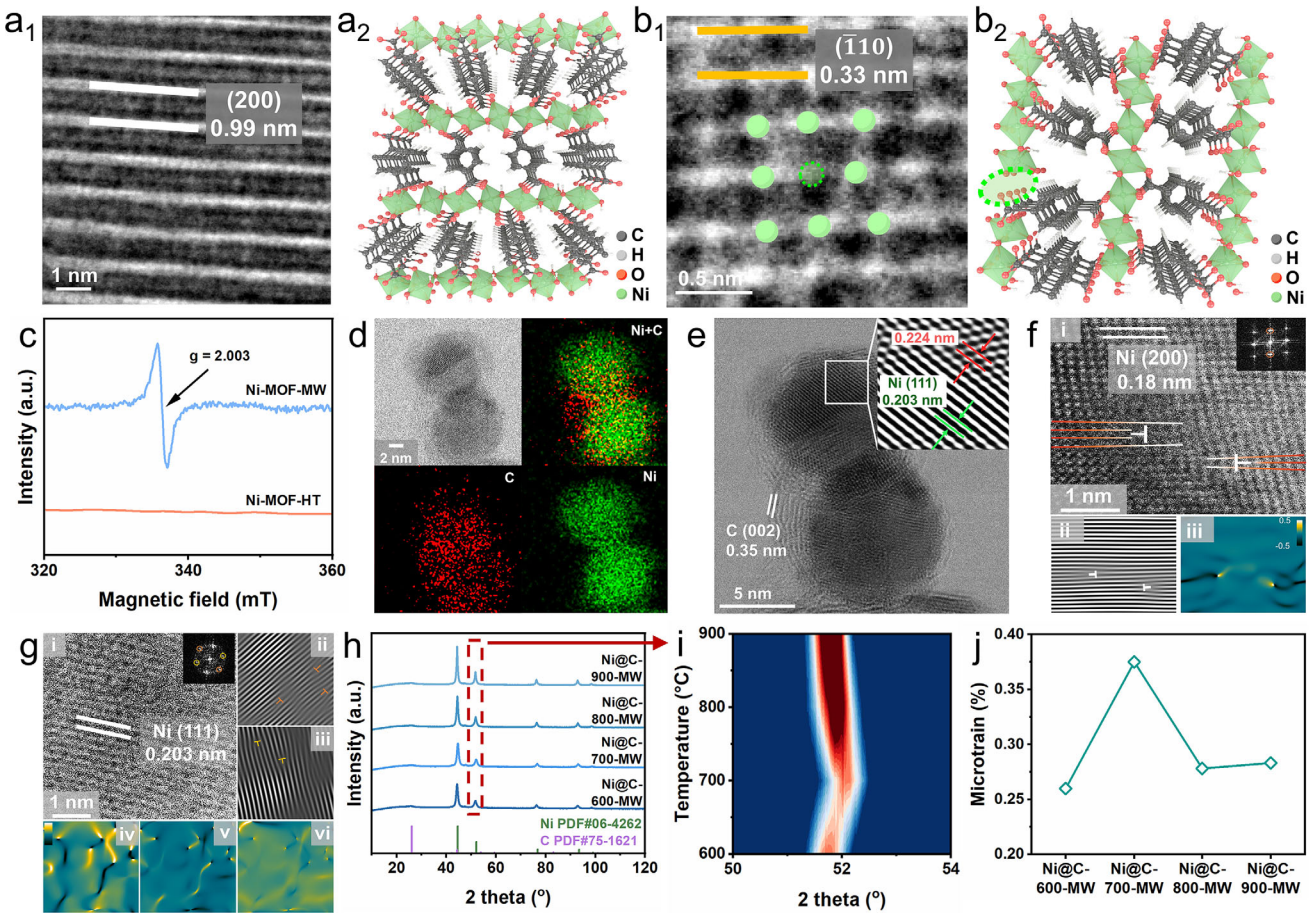
Structural and defect characterization of Ni‐MOF and Ni@C catalysts. (a, b) iDPC‐STEM images of Ni‐MOF‐MW viewed along [100] direction (a) and [110] direction (b), showing the atomic lattice structure, crystallographic planes and defect regions. (c) EPR signals for Ni‐MOF‐MW and Ni‐MOF‐HT, with the signal centered at *g* = 2.003 observed only in Ni‐MOF‐MW. (d) HAADF‐STEM image and EDX maps of Ni@C‐700‐MW, confirming the core‐shell architecture. (e) HRTEM image of Ni@C‐700‐MW showing the carbon shell and Ni core, with local Ni lattice expansion from 0.203 to 0.224 nm. (f) iDPC‐STEM image and corresponding strain mapping of Ni@C‐700‐MW along [100], with the FFT inset, inverse FFT (ii), and GPA strain map (iii). (g) HAADF‐STEM image of Ni@C‐700‐MW alone [111] (i), with inverse FFTs (ii, iii), and corresponding GPA strain maps ε_xx_ (iv), ε_yy_ (v), and ε_xy_ (vi). The⊥symbols denote additional half‐planes. (h) XRD patterns of Ni@C‐MW series obtained after pyrolysis at 600°C–900°C, with reference positions for fcc Ni (PDF#06‐4262) and graphitic carbon (PDF#75‐1621). (i) Two‐dimensional contour plot of the Ni (111) reflection for Ni@C‐MW. (j) Calculated microstrain of Ni@C‐MW catalysts obtained from WH analysis.

Thermogravimetric analysis (TGA) under oxygen atmosphere further provides quantitative analyses, yielding a linker‐to‐metal ratio of 1.25 of Ni‐MOF‐MW versus 0.93 for Ni‐MOF‐HT, confirming abundant missing cluster defects (Note S1, Figure S6 and Table S3). Furthermore, the defective Ni‐MOF‐MW experiences a two‐step decomposition profile at 430°C and 550°C, whereas Ni‐MOF‐HT collapses in a single step near 420°C (Figure S7). Such staged thermal behavior of Ni‐MOF‐MW reflects heterogeneous coordination environments associated with defect sites. The electronic signatures of missing‐cluster defects were detected by electron paramagnetic resonance (EPR) spectroscopy. Ni‐MOF‐MW shows a *g* ≈ 2.003 signal, attributable to the unpaired electrons localized at ligand vacancies surrounding the missing clusters, while the signal was silent for the highly crystalline Ni‐MOF‐HT (Figure 2c) [38].

The Ni K‐edge x‐ray absorption near‐edge structure (XANES) spectrum of Ni‐MOFs exhibits a higher absorption edge compared to that of NiO, suggesting an average Ni valence slightly higher than +2 (Figure S8a). In NiO_6_ octahedral structures of Ni‐MOFs, Ni_1_ is combined with two O and four OH atoms from two BDC ligands, while Ni_2_ is coupled with four O and two OH atoms from four BDC [39, 40]. Fourier‐transformed extended x‐ray absorption fine structure (EXAFS) fitting yields a first‐shell N─O coordination of 5.9 ± 0.9 for Ni‐MOF‐MW versus 6.3 ± 1.0 for Ni‐MOF‐HT, together with a larger Debye–Waller factor, confirming the presence of under‐coordinated Ni sites in Ni‐MOF‐MW (Figure S8b–d, Table S4) [41]. Collectively, these compositional, thermal, electronic, and local structural analyses results demonstrate that microwave‐assisted synthesis effectively introduces abundant coordination defects into Ni‐MOF‐MW, which serve as the structural origin of the strain‐rich and defective Ni@C‐MW composite upon pyrolysis.

High‐angle annular dark‐field (HAADF) STEM images, energy dispersive x‐ray spectrometry (EDX) maps, and SEM/TEM images reveal that Ni nanoparticles are uniformly encapsulated in multilayer graphitic carbon, forming distinct hetero‐Ni@C interfaces along a rod‐like carbon matrix inherited from Ni‐MOF‐MW (Figure 2d–g, Figures S9 and S10). Localized lattice expansion of Ni (111) planes from 0.203 to 0.224 nm, along with the slightly larger (002) spacing in graphitic carbon (0.35 nm) compared to well‐ordered graphite (0.33 nm), indicate increased strain accumulation in the Ni core and structural disorder of the carbon shells (Figure 2e, Figure S11). Atomic resolution imaging further provides direct evidence of structural defects. Inverse FFTs based on the (200) reflections of Ni reveal typical edge dislocations, evidenced by the insertion of half atomic planes in this area (Figure 2f‐ii). Geometric phase analysis (GPA) of the HAADF‐STEM images reveals localized strain dipoles surrounding these dislocation cores, with the ε_xy_ maps displaying negative values indicative of compressive strain within the Ni lattice (Figure 2f‐iii). Similarly, the (111) zone image again displays extra half‐plane insertions and multiple dipolar strain fields (Figure 2g), confirming that edge dislocations permeate multiple slip systems rather than being confined to a single crystallographic direction. These observations reveal that missing cluster defects facilitate early nucleation of a dense edge dislocation network across multiple lattice planes in the Ni core, introducing both long‐range strain fields and interfacial distortions.

The Ni@C composite structure is further confirmed by XRD analysis, with diffraction patterns for all Ni@C catalysts matching well with fcc Ni (PDF 06–4262) and graphitic carbon (PDF 75–1621) (Figure 2 h,i, Figure S12). For Ni@C‐HT derived from Ni‐MOF‐HT, the Ni (200) peak at around 52° progressively shifts to higher 2*θ* as temperature rises, indicating gradual lattice contraction due to particle coarsening (Figure S12a,b). In contrast, Ni@C‐MW exhibits a blue‐shift of the Ni peak at intermediate rising temperatures, followed by a sharp red‐shift at 700°C signaling a nonmonotonic strain evolution (Figure 2h,i). This atypical lattice behavior indicates that microwave‐induced precursor defects are translated into metastable strain states within the Ni cores during carbonization. To quantify these changes, Rietveld refinement and Williamson‐Hall fitting on the XRD data were performed (Figure S13). Results showed that Ni@C‐700‐MW exhibited the largest microstrain, the most negative relative lattice parameter (Δa/a), and the maximal peak broadening (FWHM) at 700°C among the series (Figure 2j, and Figure S14) [42, 43].

Graphene growth dynamics further explain the stabilization of the observed defect topology. A previous study shows that single‐layer graphene forms on Ni between 500°C and 650°C, whereas multilayer graphene domains that are incommensurate with the Ni (111) lattice nucleate above 650°C [35]. During pyrolysis, contraction of the graphitic shell imposes compressive stress that multiplies and stabilizes the dislocations in the Ni core. As a result, a high‐density multidirectional dislocation architecture becomes locked into Ni@C‐700‐MW. Spectroscopic analysis further clarifies this strain‐stabilized structural evolution. XANES and EXAFS reveal that the Ni─Ni coordination number (CN) of Ni@C‐MW catalysts increases from 11.6 ± 0.6 to 12.1 ± 0.6 as the pyrolysis temperature rises from 600°C to 900°C (Figure S15 and Table S5), indicating progressive structural ordering due to grain growth and strain relaxation [44], consistent with the XRD refinement results. Notably, Ni@C‐700‐MW remains in a slightly under‐coordinated state at 11.6 ± 0.5, while Ni@C‐700‐HT approaches the ideal value of 12.0 ± 0.6 (Figure S16 and Table S5), indicating persistent lattice disorder inherited from microwave‐derived defects for Ni@C‐700‐MW. X‐ray photoelectron spectroscopy (XPS) confirms that Ni species in all samples remain metallic, with Ni@C‐700‐MW displaying the largest negative binding energy shift, indicating electron‐rich Ni sites arising from defects, interfacial strain and potential charge transfer from adjacent defective carbon shell (Figure S17). Above 700°C, the Ni 2p_3/2_ peak shifts toward the bulk Ni value, suggesting relaxation of interfacial strain and reordering of the Ni surface.

The degree of carbon graphitization is further assessed by Raman spectroscopy. Typically, the D band (∼1350 cm^−1^) is associated with disordered carbon, while the G band (∼1580 cm^−1^) corresponds to graphitic carbon [22]. The *I*_D_/*I*_G_ ratio reaches a maximum of 1.20 for Ni@C‐700‐MW among all Ni@C‐MW and Ni@C‐HT samples (Figure S18), suggesting a defect‐rich carbon framework. To provide an integrated view, the compositional, electronic, structural, and spectroscopic metrics of the precursors and derived catalysts were compiled into defectiveness heatmaps (Figure S19). Ni@C‐700‐MW was consistently identified as the most defective catalyst across all descriptors, confirming inheritance of the defect‐rich characteristics of Ni‐MOF‐MW. Together, the strained Ni cores and defective graphitic shells form a highly distorted metal–carbon interface in Ni@C‐700‐MW, holding great promise for superior dielectric polarization and microwave energy dissipation to generate localized hot spots and improve catalytic oxidation performance [45].

When the electromagnetic waves interact with a material, a portion of the incident energy is stored while the rest is dissipated as heat [46]. Reflection loss (*R*_L_) and the effective absorption bandwidth (EAB), which represents the bandwidth where *R*_L_ < −10 dB, are therefore key parameters for evaluating microwave absorption performance [22, 47]. Normally, a more negative *R*_L_ indicates stronger absorption, with *R*_L_ values below −10 dB corresponding to over 90% microwave absorption. As shown in Figure 3a–d, Ni@C‐700‐MW achieved the smallest *R*_L_ value (−35.9 dB) and widest EAB (5.1 GHz), highlighting its superior microwave absorption capacity. Microwave attenuation ability determines how well Ni@C dissipates the absorbed microwave energy as heat, preventing excessive reflection or penetration [22], and the attenuation constant (α) was introduced to evaluate attenuation ability (Equation S8). Among all catalysts, Ni@C‐700‐MW exhibits the highest α value (Figure 3e) among the four materials pyrolyzed at different temperatures, indicating the most efficient internal energy dissipation.

**FIGURE 3:**
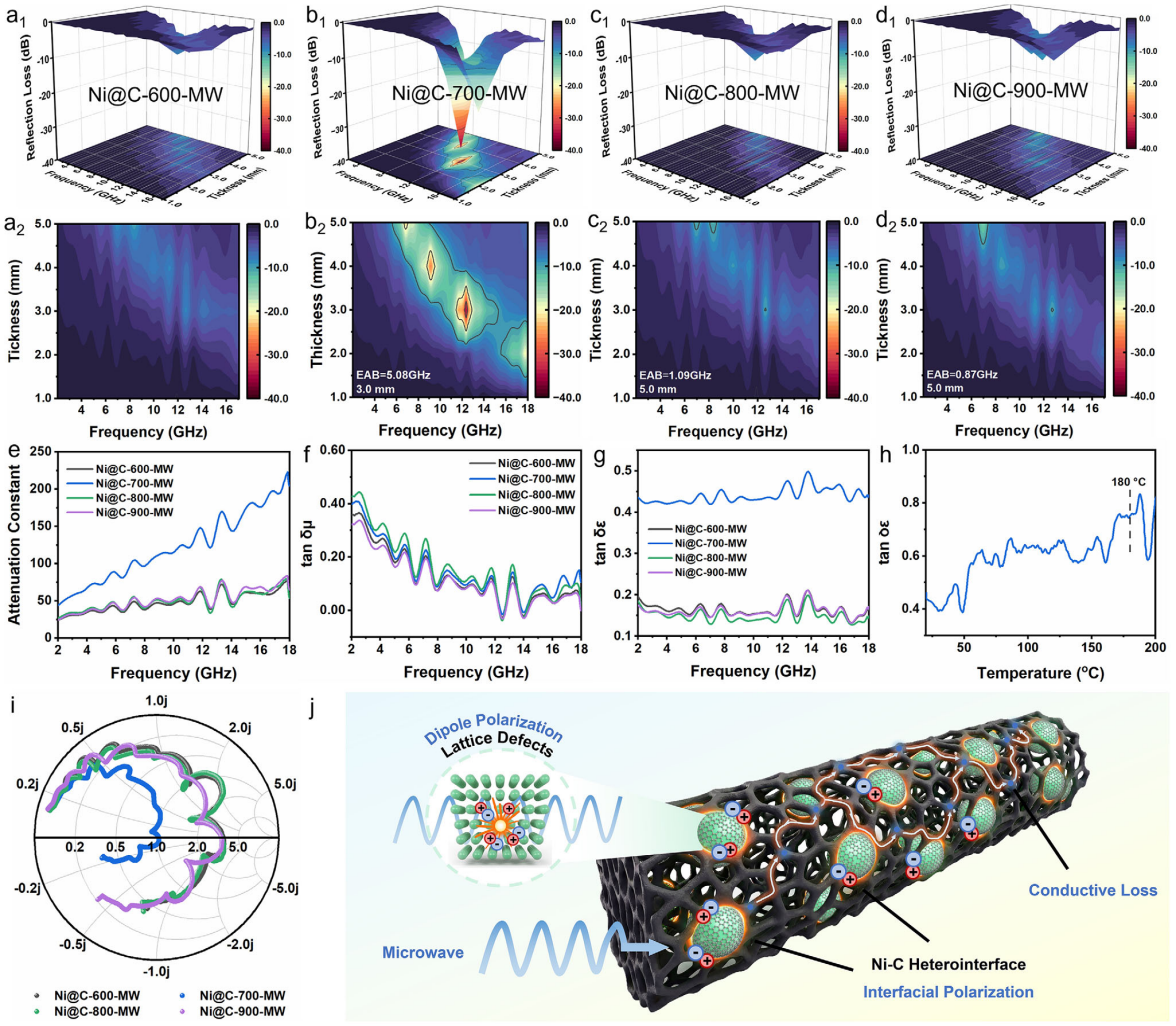
Microwave responsive capacity of Ni@C catalysts. (a_1_‐d_1_) Three‐dimensional reflection loss and a_2_‐d_2_, corresponding two‐dimensional contour maps of Ni@C‐MW catalysts. (e) Attenuation constant (α), (f) magnetic loss tangent (tan δµ) and (g) dielectric loss tangent (tan δε) of Ni@C‐MW catalysts. (h) Temperature‐dependent tan δε of Ni@C‐700‐MW. (i) Smith chart for the Ni@C‐MW series. (j) Schematic illustration of the microwave responsive mechanisms in the Ni@C‐700‐MW, including dipole polarization at lattice defects, interfacial polarization at the Ni‐C heterointerface, and conductive loss within the carbon matrix.

Analysis of loss mechanisms reveals that magnetic loss (tan δµ = µ″/µʹ) is comparable across all samples, whereas dielectric loss (tan δε = ε″/εʹ) is markedly enhanced in Ni@C‐700‐MW and increases further with temperature (Figure 3f–h). This behavior indicates that dielectric loss is the dominant contributor to microwave attenuation in the Ni@C‐MW series [11]. To further elucidate the dielectric response of the Ni@C‐MW series, the complex impedance and relaxation behavior were analyzed using the Smith chart (Figure 3i) and Cole–Cole plots (Figure S20). The Smith chart shows that the Ni@C‐700‐MW exhibits an impedance trajectory closest to the central point (*Z* = 1.0), indicating an optimized impedance match that facilitates maximum electromagnetic wave entry [46]. The Cole–Cole plots, where a series of distinct semicircular loops denotes multiple Debye relaxation events [48]. These features arise from the synergistic effects of interfacial polarization at heterogeneous Ni─C boundaries and dipole polarization induced by lattice defects. Furthermore, the characteristic upward tails point to conductive loss. Static magnetic measurements (Figure S21) confirm a stable ferromagnetic state, with saturation magnetization (Ms) and coercivity (Hc) values reflecting the structural integrity of the Ni cores. The exceptional dielectric response of Ni@C‐700‐MW originates from its defect‐engineered microstructure and optimized impedance characteristics (Figure 3j). Specifically, pronounced microstrain, dense edge dislocations, and abundant Ni─C heterointerfaces act as polarization centers, forming a complex network of dipolar and interfacial relaxation that efficiently traps electromagnetic energy. Simultaneously, the conductive graphitic shell provides electron‐transport pathways, while permittivity contrasts at coherent Ni─C interfaces improve impedance matching, enabling deeper microwave penetration. These synergistic effects trap electromagnetic energy within the catalyst and promote efficient conversion into localized heat, thereby endowing Ni@C‐700‐MW with superior microwave responsiveness.

### 2.2 HDPE Degradation by the Microwave‐Assisted Fenton‐Like System

As shown in Figure 4a, bulk microwave‐induced thermal degradation alone led to 17.5 wt% weight loss of HDPE (commercial microplastic‐sized particles, average size 164 µm) after 12 h at 180°C; this value increased to 32.4 wt% when PMS was introduced into the MW‐PMS system. Further introducing Ni@C catalysts improved degradation efficiency to 45.4–62.9 wt%, with Ni@C‐700‐MW reaching the highest efficiency of 62.9 wt%, substantially outperforming both Ni@C‐700‐HT (50.1 wt%) and other Ni@C‐MW catalysts with less microwave responsiveness. Across the Ni@C‐MW series, the MOF‐templated rod‐like morphology is retained (Figure S9), while the Ni core size increases monotonically with pyrolysis temperature (Figure S10). The observed catalytic activity, therefore, cannot be attributed to differences in morphology or particle size, but instead correlates with the defect‐governed microwave response of the catalysts (Figure 3). This is supported by the positive correlation of multiple defect descriptors with both dielectric loss and degradation efficiency across the series (Figure S22). Microstrain shows the strongest correlation, indicating that defects within the Ni core play a key role in the enhanced dielectric loss and catalytic performance of Ni@C‐700‐MW.

**FIGURE 4:**
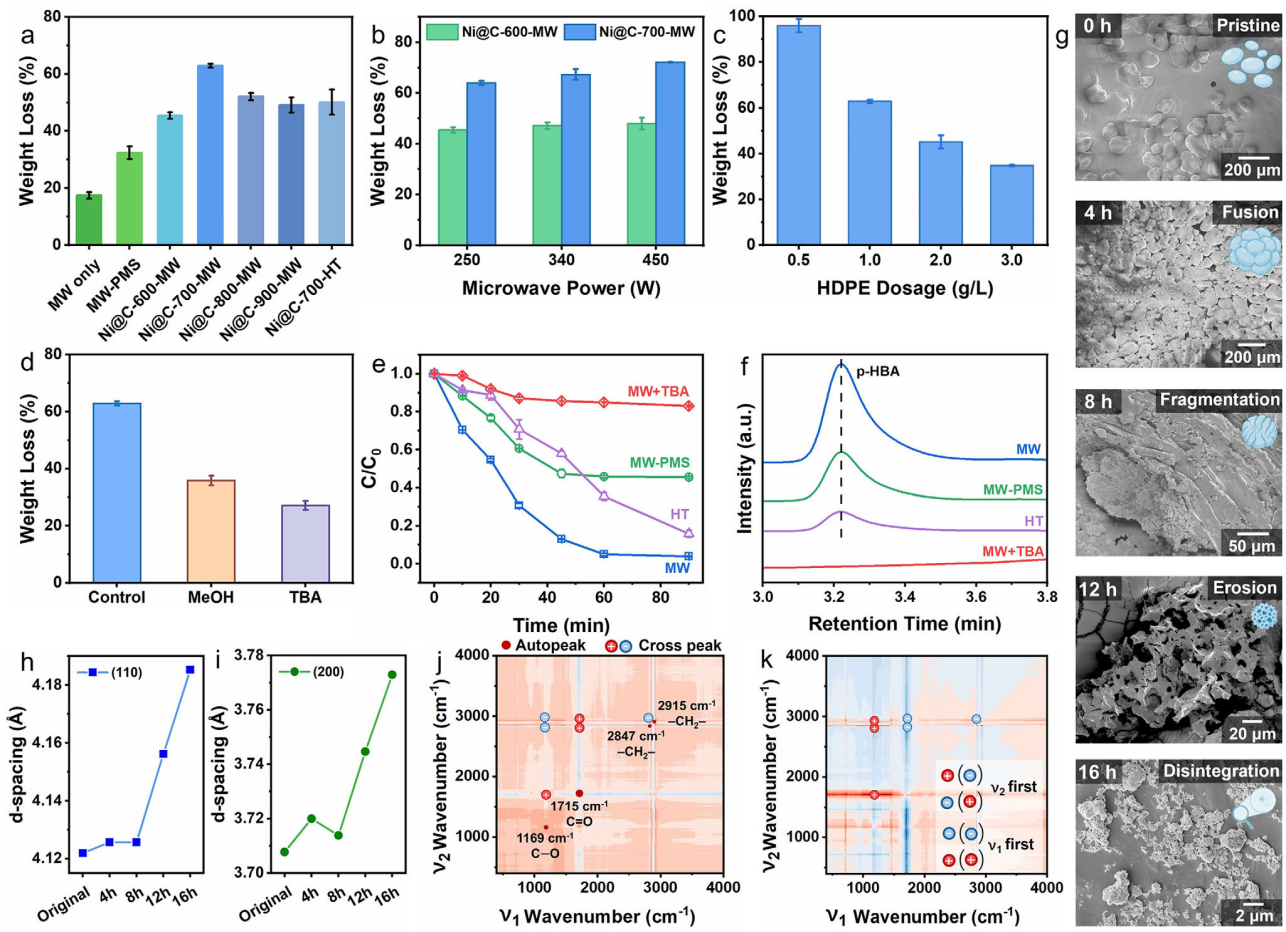
Performance and mechanistic characterization of HDPE degradation. (a) Weight loss of HDPE using various Ni@C‐MW catalysts, compared with the Ni@C‐700‐HT. (b) Effect of microwave power and (c) HDPE dosage on degradation efficiency. (d) Quenching experiments using MeOH and TBA to identify active species. (e) Degradation kinetics of BA under the MW, MW‐PMS, and HT systems, including the MW system with TBA addition. (f) HPLC chromatograms of p‐HBA formation from BA oxidation. (g) Time‐resolved SEM images and schematics showing the structural disintegration of HDPE from pristine particles to fragmented residues (0–16 h) in the MW system. (h, i) Lattice d‐spacing evolution for the (110) and (200) planes of HDPE during the reaction. 2D‐COS synchronous (j) and asynchronous (k) FT‐IR spectra of HDPE during 0–16 h reaction. All reactions used [catalysts] = 0.1 g/L, [PMS] = 4 g/L, temperature = 180 °C, pH 2.4, microwave power = 250 W in MW system unless otherwise specified. Error bars represent standard deviations from triplicate experiments.

Three primary reaction systems were defined to evaluate the collaborative effects of catalysts and the external energy mode (Figure S23 and Table S1): a noncatalytic microwave‐activated PMS system (MW‐PMS), a microwave‐assisted catalytic system combining Ni@C‐700‐MW and PMS (MW), and a conventional hydrothermal system utilizing Ni@C‐700‐MW and PMS in the oven (HT). Under identical conditions, the optimal Ni@C‐700‐MW achieved only 48.5 wt% degradation under hydrothermal conditions (Figure S24a), indicating the critical role of microwave irradiation in intensifying surface‐localized reactions for plastic decomposition and PMS activation. Pseudo‐first‐order kinetic analysis of the degradation profiles gives an initial apparent rate constant of 0.067 h^−1^ for the MW system (Figure S24b), approximately 1.4 times that of the HT system (0.047 h^−1^) and nearly four times that of the MW‐PMS system (0.017 h^−1^). This result indicates that enhanced HDPE degradation requires the combined contributions of Ni@C‐700‐MW, PMS, and microwave irradiation, and cannot be explained by conventional thermal heating or PMS self‐activation by microwave alone. Control experiments using commercial graphite and Fe_3_O_4_, two representative microwave absorbers with low defect densities [49], resulted in only 38.8 and 27.9 wt% HDPE degradation, respectively (Figure S25), indicating that microwave responsiveness alone is insufficient to lead to the superior activity of Ni@C‐700‐MW. Considering the strong microwave attenuation and dielectric loss of Ni@C‐700‐MW, together with literature reports of catalyst‐associated local hotspot heating under microwave irradiation, even with bulk temperature control [50, 51], transient local microwave energy concentration near microwave‐responsive Ni@C‐700‐MW is expected. Based on the heat transfer model, the interfacial temperature gradient scales positively with catalyst dielectric loss and microwave intensity, but decreases with the thermal conductivity of the surrounding medium [52]. Therefore, local energy dissipation is more likely to accumulate in the catalyst‐HDPE region, where HDPE has a lower thermal conductivity (0.4 W m^‒1^ K^‒1^) than the PMS solution (0.68 W m^‒1^ K^‒1^) [53]. Increasing microwave power from 250 to 450 W further improved the degradation efficiency of HDPE from 62.9 to 72 wt% in the MW system (Figure 4b), whereas the poorly responsive Ni@C‐600‐MW showed only a 2.7 wt% improvement. This material‐dependent response suggests that the highly microwave‐responsive Ni@C‐700‐MW enables more efficient conversion of increased power input into localized thermal energy for catalytic plastic oxidation. Reducing initial HDPE dosage to 0.5 g/L unlocked a peak degradation of 96 wt% (Figure 4c). Additional optimization confirmed that prolonged reaction times, elevated temperatures, and acidic conditions collectively favor degradation (Figure S24). Ni@C‐700‐MW retained approximately 67%–69% of its activity at pH 5.0–7.0, indicating effective plastic degradation under near‐neutral conditions (Figure S24d). Ultimately, to balance high efficiency with realistic microplastic concentrations and resource conservation, the standard parameters were established as follows: 0.1 g/L Ni@C‐700‐MW, 1.0 g/L HDPE, 4.0 g/L PMS, 250 W, 12 h, 180°C, and pH 2.4.

To elucidate the reactive species in the MW system, comprehensive kinetic and analytical approaches were conducted, including competitive radical quenching, chemical probe analysis, electron paramagnetic resonance (EPR), and open circuit potential (OCP) measurements. Radical quenching experiments were conducted using *tert*‐butanol (TBA, 500 mM) and methanol (MeOH, 1000 mM) with optimized concentrations to allow complete radical scavenging (Figure S27). MeOH is a classic scavenger for both •OH and SO_4_^•−^ ($k_{•OH,\;MeOH}=9.7\times 10^{8}\;M^{-1}\;S^{-1};\;\;k_{SO_{4}^{{•}^{-}},\;MeOH}=3.2\times 10^{7}\;M^{-1}\;S^{-1}$) [54], while TBA effectively quenches •OH selectively ($k_{•OH,\;TBA}=5.9\times 10^{8}\;M^{-1}\;S^{-1};\;\;k_{SO_{4}^{{•}^{-}},\;TBA}=8.0\times 10^{5}\;M^{-1}\;S^{-1}$) [55]. TBA and MeOH suppressed degradation efficiency from 62.9% to 27.1% and 35.8%, respectively (Figure 4d). Notably, TBA caused stronger inhibition despite its lower reaction rate with •OH. This counterintuitive behavior is consistent with the short half‐life of •OH (2 × 10^−8^ s), which restricts its diffusion from the generation site [56]. The greater hydrophobicity of TBA promotes its preferential partitions near the Ni@C‐700‐MW/HDPE interface, allowing it to more effectively compete with HDPE for surface‐bound •OH than MeOH [55].

Benzoic acid (BA) was used as a featured chemical probe, which can be selectively converted to p‐hydroxybenzoic acid (p‐HBA) by •OH [57]. Quantification of p‐HBA formation confirms that the MW system produces markedly higher levels of •OH than either the MW‐PMS or HT system (Figure 4e,f). During the initial 20 min, MW‐PMS also exhibited faster BA degradation and greater p‐HBA production than the HT system, indicating more rapid early‐stage PMS activation. This enhancement likely arises from the rapid microwave‐induced polarization of the peroxide O─O bonds and accelerated bulk solution heating [58]. Furthermore, the negligible p‐HBA formation upon the addition of TBA validates probe specificity and supports the dominant role of •OH, consistent with the surface‐confined reactivity suggested by the quenching experiments. EPR measurements detected both •OH and SO_4_^•−^ species, with the highest signal intensity observed for Ni@C‐700‐MW (Figure S28a). In contrast, no signal was observed for the TEMP‐^1^O_2_ or the DMPO‐^•^O_2_ under the same conditions (Figure S28b), indicating that ^1^O_2_ and ^•^O_2_^−^ are not major reactive species in this system. The quenching experiment for ^1^O_2_ and ^•^O_2_^−^ using furfuryl alcohol (FFA) and p‐benzoquinone (p‐BQ) produced only weak inhibition, lowering the degradation efficiency to 45.8% and 41.1%, respectively (Figure S28c), much less than with TBA or MeOH. Since FFA and p‐BQ can both scavenge radicals and react with PMS directly, their limited effect is consistent with scavenging of the dominant •OH and SO_4_^•−^ rather than with a major nonradical pathway [59]. The PMS consumption test confirmed that PMS was effectively activated (Figure S29). OCP analysis revealed that Ni@C‐700‐MW possesses the lowest initial surface potential and largest PMS‐induced shift (Figure S28d), indicating enhanced interfacial electron transfer capability. Together, these results support that the composite‐induced electron‐rich surface of Ni@C‐700‐MW facilitates PMS activation and promotes selective hydroxyl radical generation under microwave irradiation.

The structural and molecular evolution of HDPE was monitored from the macroscopic to the molecular scale using SEM, XRD, Fourier transform infrared spectroscopy (FT‐IR), and two‐dimensional correlation spectroscopy (2D‐COS). SEM images illustrate the progression from surface pits around the catalyst and melted agglomerates to the development of holes and eventual fragmentation into smaller, rougher particles (Figure 4g and Figure S30). The initiation of surface pitting at discrete catalyst‐polymer contact points is consistent with localized heat and catalytic activation at the Ni@C‐700‐MW interface. XRD corroborates this sequence by revealing anisotropic structural changes (Figure 4 h,i and Figure S31). The (200) reflection monitors chain‐axis coherence while the (110) reflection tracks lateral packing stability [60]. During the initial 4 h, both (110) and (200) reflections exhibit increased interplanar spacing, consistent with early structural disordering and possible chain scission under localized heat and radical attack. From 4 to 8 h, the (200) spacing decreases while the (110) reflection remains relatively stable, indicating the polymer chain reorganization within partially disrupted lamellar domains. This likely reflects local densification after interfacial packing disruption, potentially involving increased chain tilt that enables more efficient lateral packing [15, 60]. Beyond 8 h, a sharp spacing increase across both planes marks a progressive loss of crystallinity order under sustained thermochemical breakdown.

FT‐IR and 2D‐COS provide molecular‐level insight into bond evolution. FT‐IR shows the emergence of C═O (1712 cm^−1^) and C─O (1168 cm^−1^) bands within 4 h, with the carbonyl index (CI) rising from 0.12 to 0.32, indicating the onset of polymer oxidation (Figure S32) [15, 61, 62]. Continued reaction leads to progressive oxidation, with CI surpassing 0.5 by 12 h and reaching 0.78 at 16 h. Synchronous 2D‐COS spectra identify four prominent autopeaks at 1169, 1715, 2847, and 2915 cm^−1^, positioned on the power spectrum diagonal for all three systems (Figure 4j, Figures S33c and S34c), corresponding to C─O stretching, C═O stretching, CH_2_ symmetric stretching, and CH_2_ asymmetric stretching, respectively [62, 63]. The presence of auto‐peaks at these wavenumbers confirms that all four species actively respond to the progressive oxidation, with their intensities directly reflecting the magnitude of spectral change at each wavenumber across reaction timepoints. The MW system exhibits higher auto‐peak intensities than MW‐PMS or HT systems, confirming that microwave‐assisted catalytic degradation induces more intensive disruption of polymer C─H bonds and synchronous formation of oxidative functional groups than noncatalytic ROS or conventional thermal pathways. Based on Noda's rules [64], combined synchronous and asynchronous correlation maps can reveal the change order of specific chemical bonds Note S4 and Table S6–S8). In the MW system, changes occur in the order of 2915 cm^−1^ > 2847 cm^−1^ > 1169 cm^−1^ > 1715 cm^−1^, indicating that chain activation and hydrogen abstraction precede oxidative functionalization. In contrast, the HT system follows 1169 cm^−1^ > 1715 cm^−1^ > 2847 cm^−1^ > 2915 cm^−1^, while MW‐PMS follows 1715 cm^−1^ > 1169 cm^−1^ > 2847 cm^−1^ > 2915 cm^−1^, both following oxidation‐first pathways, where carbonyl formation precedes, accompanied by significant backbone breakage. This inversion of reaction order demonstrates that localized hotspot concentrated radical attack at the Ni@C‐700‐MW catalyst interface preferentially activates disordered polymer chain segments prior to oxidative functionalization.

We also systematically evaluated carbon mass balance, product selectivity, fuel‐range valorization and gas evolution, to link the nanoscale degradation mechanism with macroscopic upcycling performance. The MW system at 0.5 g/L HDPE loading (denoted as MW‐0.5), which achieves near‐complete polymer degradation, was specifically incorporated into the analysis of valuable product distribution and yield. All values are reported in milligrams of carbon (mg C), with initial loadings of 42.8 mg C for 1.0 g/L HDPE and 21.4 mg C for 0.5 g/L HDPE. The carbon distribution across unreacted solid residues, liquid products, and gaseous species was determined by gravimetric analysis, total organic carbon (TOC) quantification, and mass difference calculations (see Note S5 for detailed calculations). Time‐resolved carbon tracking reveals a continuous phase transfer from solid polymer to fluid products. In the MW system, gas formation (14.1 mg C) exceeds liquid accumulation (2.17 mg C) at 4 h (Figure 5a), indicating that early degradation is dominated by rapid chain scission coupled with oxidation, favoring the direct formation of gaseous products before substantial liquid‐phase product accumulation. Between 8 and 12 h, liquid product accumulation becomes more prominent as chain scission extends into more ordered domains, while gas production continues through concurrent oxidative conversion of both primary fragments and intermediate species.

**FIGURE 5:**
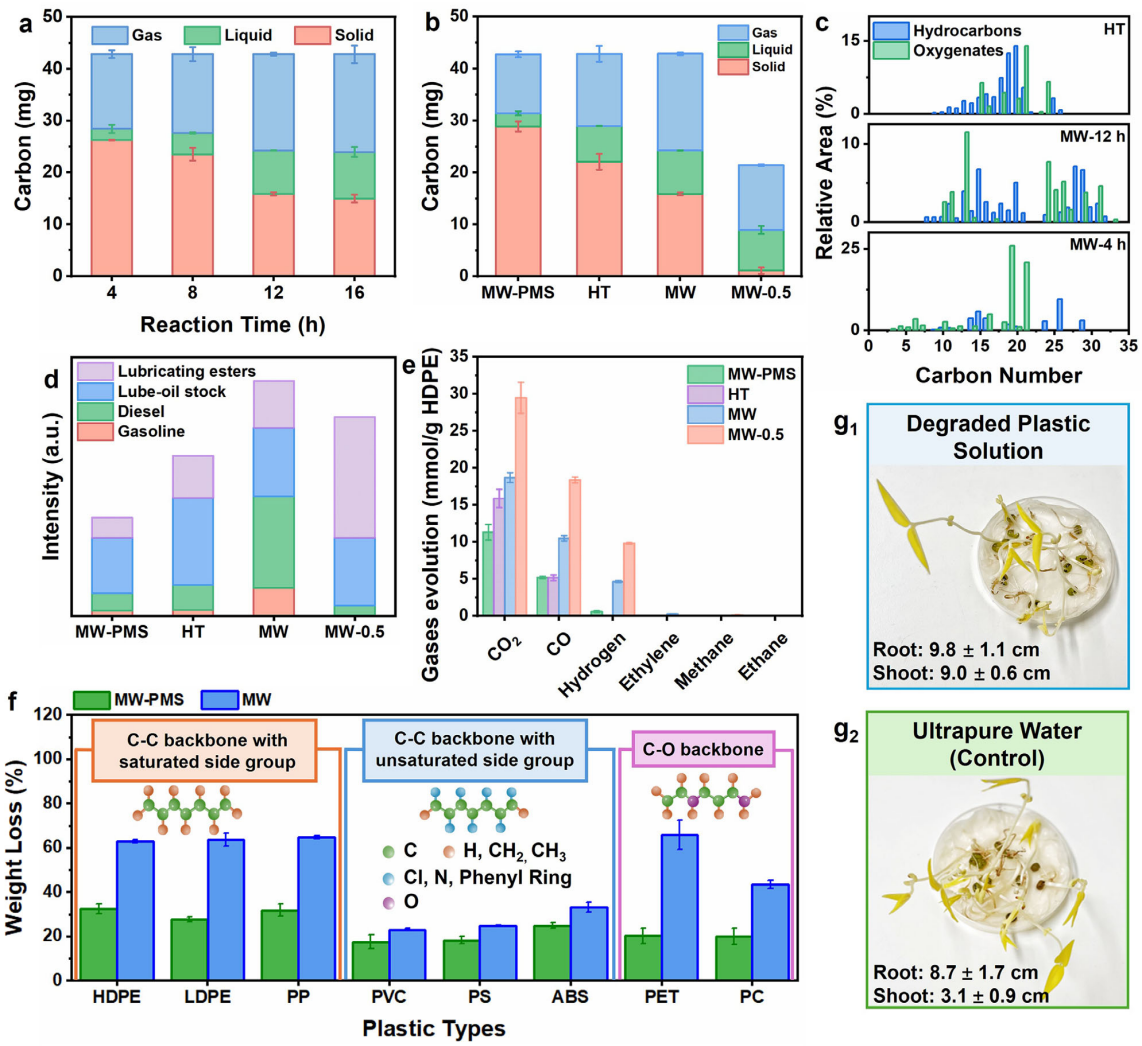
Carbon balance, product distribution, and universality. (a) Time‐dependent carbon distribution in gas, liquid, and solid phases during HDPE degradation in the MW system. (b) Carbon distribution across different systems (MW‐PMS, HT, and MW) and at a lower HDPE dosage (MW‐0.5). (c) Carbon number distribution of hydrocarbons and oxygenates in the liquid phase for HT, MW‐12 h, and MW‐4 h systems. (d) Distribution of valuable liquid products. (e) Gas evolution rates for major gaseous products across different systems. (f) Universality of the MW system for the degradation of common plastic types, categorized by their backbone structure. (g) Phytotoxicity evaluation showing the growth of mung bean seedlings in degraded plastic solution (g_1_) compared to a control of ultrapure water (g_2_). All reactions were conducted at 180°C and initial pH 2.4 with [PMS] = 4 g/L. Catalytic systems MW, MW‐0.5 and HT used [Ni@C‐700‐MW] = 0.1 g/L. [HDPE] was 1.0 g/L except for MW‐0.5 at 0.5 g/L. Microwave systems (MW, MW‐0.5, and MW‐PMS) used 250 W power while the HT system utilized conventional oven heating. Error bars represent standard deviations from triplicate experiments.

Lower HDPE loading in the MW‐0.5 system leads to distinct product evolution and distribution. Specifically, liquid intermediates accumulate rapidly within the first 4 h, nearly complete solid decomposition is achieved by 12 h, and continued gas formation occurs at extended reaction times (Figure S35a). This shift arises from the increased catalyst‐to‐substrate ratio, which enhances heat and radial flux per unit polymer, leading to faster chain scission and transient accumulation of liquid intermediates before further conversion. In contrast, the noncatalytic MW‐PMS system retains a dominant solid carbon fraction throughout the reaction, producing only 1.45 mg C of liquid hydrocarbons after 16 h (Figure S35b), indicating kinetically limited bond cleavage. The conventional HT system shows a different pathway. Liquid products accumulate initially and peak at 10.03 mg C at 8 h, followed by a decline due to thermally driven overcracking (Figure S35c). This process facilitates the conversion of liquid intermediates into gas, which reaches a cumulative yield of 20.26 mg C by 16 h. Direct comparison at 12 h (Figure 5b) shows that the MW system achieves deeper polymer conversion and higher liquid retention than both the MW‐PMS and HT systems.

To further probe the underlying mechanism, carbon number distributions were analyzed (Figure 5c, Figures S36 and S37). The degradation behavior can be interpreted within a framework of a heterogeneous mobility field in the constrained polymer network. Detailed spectral analysis (Figure 5c) reveals that the MW system produces a narrow distribution of initial products dominated by C_19_‐C_21_ oxygenated species at 4 h. The specific carbon number range serves as a chemical fingerprint of the physical polymer topology. Because the densely packed crystalline lamellae are robust against radical attack during the initial stage, the oxidation is limited to the surface molecules, loose chain segments, and amorphous domains. Consequently, the intermediate lengths statistically reflect the characteristic chain segment length of the amorphous domains. This is supported by the 2D‐COS response at 2915 cm^−1^ and the initial d‐spacing expansion observed by XRD, both of which indicate reduced packing constraints and enhanced accessibility in amorphous regions relative to crystalline domains. The resulting fragments are expected to exhibit restricted segmental mobility within their local environments, leading to prolonged residence times and increasing the probability of further functionalization prior to diffusion. The temporal evolution of products reflects progressive disruption of the polymer network (Figures S37a,b and S38a). At 8 h, a sharp increase in alkane intensity alongside the emergence of C_15_‐C_30_ hydrocarbons indicates the progressive extension of chain scission beyond initially more accessible amorphous regions as degradation progresses into more ordered domains. By 12 h, a broad C_5_‐C_35_ distribution is established, accompanied by peak liquid product intensity and maximum chemical diversity, featuring abundant alkanes, esters, and ethers. Detailed identification is provided in Table S9. This coincides with the collapse of crystalline ordering and extensive backbone fragmentation. It should be noted that the oxygenated products originate not only from the primary catalytic oxidation of HDPE but also from subsequent secondary intermediate transformations occurring under the reaction conditions, such as hydrolysis. Cross‐system comparison highlights distinct mechanistic pathways (Figures S37c,d and S38b).

The noncatalytic MW‐PMS system exhibits limited evolution of product distribution and minimal overall liquid intensity, with a predominance of higher carbon number species, suggesting insufficient energy input for extensive chain scission and a reaction pathway restricted to superficial oxidation (Figure S37c). In the HT system, the emergence of a C_15_‐C_22_ distribution aligns with the 2847 cm^−1^ 2D‐COS priority (Figure S34c). This suggests that under uniform thermal heating, radical activation lacks the amorphous selectivity observed under microwave conditions and instead promotes a preferential involvement of crystalline domains, where scission scales are governed by the intrinsic lamellar thickness of the polymer, yielding a liquid pool with limited oxidative diversity. In contrast, the MW‐0.5 system promotes rapid conversion of intermediate liquid‐phase fragments and a concomitant increase in gaseous products, indicating that high‐intensity catalytic hotspots promote sequential bond cleavage and overcracking at a rate that exceeds the stabilization of liquid intermediates. At 12 h, the MW system produces mainly C_9_‐C_22_ gasoline‐/diesel‐range hydrocarbons, while generating more H_2_ and light hydrocarbons (C_1_‐C_2_) than either MW‐PMS or HT systems (Figure 5d,e). This enhanced fragmentation and H_2_ evolution can be attributed to a localized catalytic hotspot, which promotes coupled dehydrogenation and bond scission processes with higher efficiency than under purely oxidative conditions or uniform thermal heating. Further quantification of C_1_‐C_4_ alkanes and alkenes showed that these species contributed only a minor fraction of the gaseous carbon in the MW and MW‐0.5 systems, with CO_2_ and CO remaining the dominant gaseous carbon products (Figure S39). Particularly, while the absolute liquid intensity in the MW‐0.5 system is reduced due to extensive gasification, its chemical profile exhibits a high proportion of oxygenates relative to alkanes (Figure S38b). This compositional transition aligns with the liquid‐phase carbon number distribution, confirming that the liquid in the MW‐0.5 system is even richer in lube‐oil stocks and lubricating esters relative to the gasoline/diesel fraction, confirming efficient carbon valorization at lower HDPE loadings (Figure 5d).

Regarding energy consumption, microwave‐assisted systems consumed substantially less energy than the conventional HT route, owing to their faster heating rates and lower power demand (Figure S26). As shown in Table S10, the MW system required only 15.3 kJ/min, compared with 25.8 kJ/min for the HT system. Based on the distribution and yield of valuable products in each system, the potential revenue for MW‐based HDPE upcycling was assessed (Table S11). The MW‐PMS and HT systems are projected to generate approximately US$111.9 and US$149.1 per ton of HDPE, while the MW and MW‐0.5 systems are expected to achieve returns of US$260.6 and US$427.5 per ton of HDPE, respectively. Further downstream costs, such as effluent neutralization or treatment of the spent aqueous phase, should be considered for practical application and will depend on the composition, buffering capacity and handling approach of the postreaction liquid. Phytotoxicity tests using mung beans showed 100% germination in the degraded solution, with root and shoot lengths exceeding those grown in ultrapure water, suggesting that the degradation products are nontoxic and can be leveraged (Figure 5g, Figure S41 and Table S12). The recyclability of Ni@C‐700‐MW has been investigated over six consecutive runs. The HDPE degradation efficiency decreased from 62.9 wt% in the first run to around 50 wt% after four runs and around 34 wt% after six runs, indicating partial reusability with gradual activity loss (Figure S42). Postreaction XRD and EXAFS analysis indicate that Ni@C‐700‐MW retained its crystal structure, but with a sharpened Ni peak and a reduced Ni─Ni coordination number in EXAFS, suggesting changes in the local Ni environment that lead to lower catalytic efficiency (Figures S43 and S44, and Table S13). ICP‐MS analysis revealed that only 0.35 wt% Ni was leached after a 12 h reaction, corresponding to 1.3% of the initial Ni content in Ni@C‐700‐MW. Applicability tests revealed that the MW system can achieve over 60 wt% degradation for PET and saturated C─C backbone plastics (e.g., LDPE and PP), while unsaturated C─C backbone plastics (e.g., PVC, PS, and ABS) were more resistant (Figure 5f). To further assess practical applicability, degradation experiments were conducted at lower concentrations and with mixed plastic streams. The MW system achieved 95 ± 1.4 wt% degradation of a 0.1 g/L HDPE within 1 h (Figure S45). A mixed plastic stream containing equal proportions of HDPE, PP, PVC, PET, and PS achieved 81 ± 2.6 wt% degradation at 0.5 g/L and 51.2 ± 2.3 wt% degradation at 1.0 g/L after 12 h. Moreover, compared with other reported systems, Ni@C‐700‐MW achieves comparable or superior degradation efficiency with only 0.1 g/L catalyst, representing an order‐of‐magnitude improvement in mass‐normalized catalytic activity (Table S14). Radical attack is governed by the combination of bond strength, polarity, resonance stabilization, steric accessibility, and the polymer's glass transition or melting temperature. In this regard, saturated C─C backbones degrade efficiently due to low steric hindrance to radical attack, low C─H bond dissociation energies, and melting‐enhanced hotspot contact, which breaks down the backbone and reduces chain length, leaving smaller segments vulnerable to radical attack [65, 66]. For PET, polar ester linkages enhance microwave adsorption and introduce susceptible sites for radical‐induced cleavage, enabling efficient degradation. In contrast, π‐conjugated aromatic structures in styrene polymer (PS, ABS) stabilize radical intermediates and suppress chain fragmentation, while the chlorination impedes cleavage [67]. Ultimately, this microwave‐assisted catalytic system is designed to operate with an integrated plastic management strategy, serving as a complementary downstream route to convert concentrated (micro) plastic residues into valuable products.

### 2.3 Mechanistic Discussion

Here, we propose a unified mechanism for HDPE degradation that integrates catalyst structure, microwave response, and reaction pathway in the MW system (Figure 6 and Figure S46). The structural integration of strained Ni cores, abundant edge dislocations, and Ni─C heterointerfaces enhances dielectric loss and interfacial polarization, enabling efficient microwave absorption and conversion into localized hotspots at the catalyst surface. These localized hotspots play three coupled roles during HDPE degradation. First, localized thermal intensification softens and disorders the HDPE crystalline lamellae, increasing chain mobility and the accessibility of the amorphous interlamellar domains. This enhanced accessibility reduces local steric constraints and facilitates preferential radical attack within these more accessible regions. Moreover, the electron‐rich catalyst surface promotes interfacial electron transfer to PMS, generating surface‐associated •OH and SO_4_^•−^ species. Simultaneously, microwave and microwave‐induced bulk heating accelerate PMS decomposition, providing an additional source of ROS that complements interfacial radical generation and sustains oxidation beyond the immediate hotspot region. Collectively, defect‐enhanced microwave absorption, hotspot‐induced structural activation, and synergistic radical generation accelerate reaction kinetics, enabling efficient HDPE degradation and concurrent formation of value‐added products.

**FIGURE 6:**
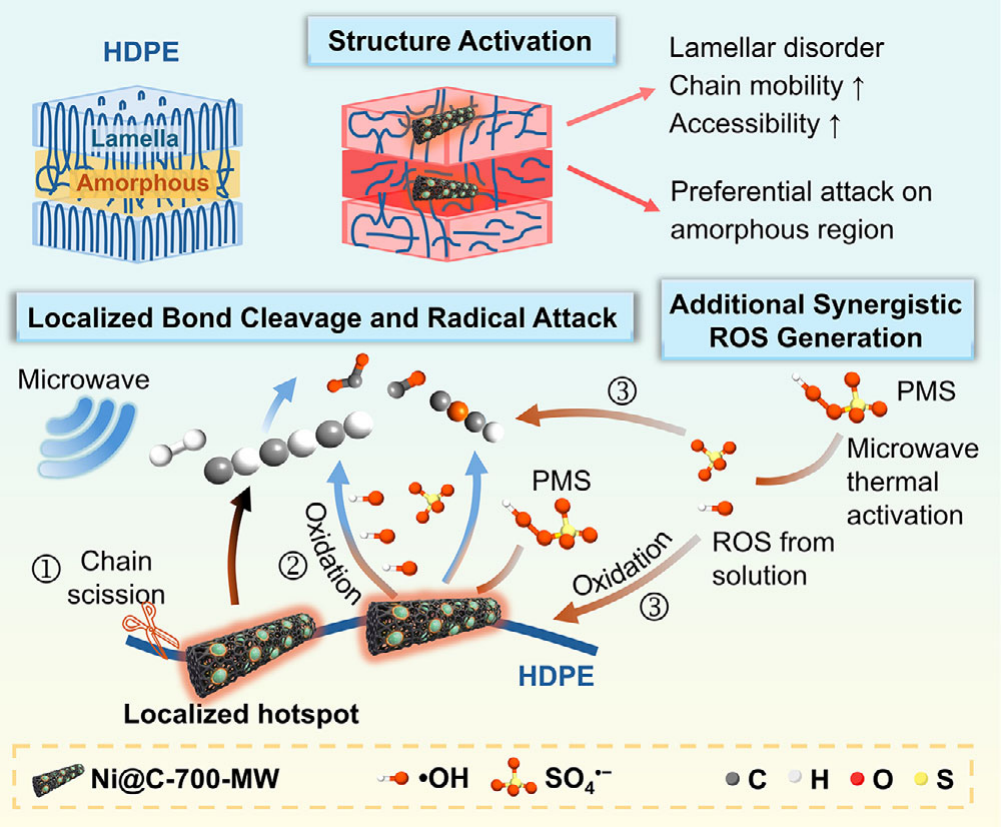
Proposed mechanism for microwave‐catalytic degradation of HDPE.

Removing either the catalyst or the localized microwave field fundamentally shifts this degradation pathway (Figure S47). In the MW‐PMS system, PMS is activated by direct microwave irradiation and bulk thermal decomposition, but the absence of localized hotspots restricts radical attack to surface oxidation. In the HT system, uniform thermal heating enables moderate catalytic PMS activation to generate ROS. However, lacking localized hotspots, polymer softening and chain accessibility are governed entirely by the bulk reaction temperature rather than targeted interfacial activation. This nonselective thermal environment drives an oxidation‐first degradation pathway, where prolonged reaction leads to the oxidative overcracking of accumulated liquid intermediates into gaseous products.

## 3 Discussion

In summary, this work demonstrates a sustainable “plastic‐to‐catalyst” and “plastic‐to‐product” strategy for the degradation and conversion of various types of plastics. Using a two‐stage defect engineering approach, waste PET bottles are upcycled into microwave‐responsive Ni@C‐MW core‐shell catalysts. Microwave‐assisted MOF nucleation generates abundant missing‐cluster defects, which are inherited during pyrolysis to produce defective Ni cores encapsulated within graphitic carbon shells. The rationally designed lattice distortions and Ni─C heterointerfaces enhance dielectric loss and interfacial polarization, driving efficient microwave energy conversion. Under microwave irradiation, this energy conversion generates localized catalytic hotspots that enhance polymer softening and chain mobility as well as interfacial accessibility while accelerating PMS activation and oxidative radical generation for HDPE degradation. This coupled activation mechanism mitigates the limitations of superficial oxidation and nonselective overcracking observed under noncatalytic and conventional heating conditions, enabling efficient HDPE degradation together with the formation of value‐added products.

## 4 Methods

### 4.1 Synthesis of Catalysts

Ni‐MOF‐MW was synthesized via a one‐step microwave‐assisted hydrothermal reaction of PET. Generally, 3.4 g Ni(NO_3_)_2_·6H_2_O was dissolved in 30 mL DMF and 15 mL H_2_O. Subsequently, 2.3 g PET were added to the mixture. The mixture was transferred to a 100 mL reactor and heated in a microwave system (Milestone ETHOS EASY) at 180°C for 24 h. The resultant Ni‐MOF‐MW was centrifuged, washed, and dried at 90°C, yielding 84.9 wt%. Ni‐MOF‐HT was prepared under identical conditions, using a conventional oven rather than a microwave.

Ni@C‐MW were obtained through direct thermal pyrolysis of Ni‐MOF. In a typical synthesis, Ni‐MOF‐MW was well ground and treated at 700°C for 3 h in an argon atmosphere. After naturally cooling to room temperature, the as‐prepared materials were ground and immersed in concentrated 1 M H_2_SO_4_, stirred for 2 h to thoroughly remove nickel compounds. The sample was then washed with DI water to neutral conditions and dried at 80°C overnight. The resulting product was denoted as Ni@C‐700‐MW. To investigate the effect of pyrolysis temperature on the structure and reactivity of the catalysts, we further prepared Ni@C‐MW at different temperatures (600°C, 800°C, and 900°C), denoted as Ni@C‐600‐MW, −800‐MW, and −900‐MW, respectively. The schematic illustrations of the catalyst synthesis are displayed in Figure 1a. Ni@C‐700‐HT was prepared from Ni‐MOF‐HT using the same procedure described above.

### 4.2 Catalytic Oxidation of HDPE

All the experiments were performed in 100 mL Teflon vessels and heated in a microwave system at set temperatures unless specifically mentioned. A given amount of Ni@C‐MW catalysts and HDPE were dispersed into 50 mL of a specific amount of PMS in a Teflon vessel. The vessel was then carefully sealed and placed in a microwave reactor at the set temperature. After the reaction, the vessel was naturally cooled down to room temperature. The catalysts were collected by magnetic separation. The degradation residues were collected by filtration through a preweighed 0.45 µm membrane, washed several times with DI water, then dried and weighed as the solid product. A simple method was adopted to measure plastic degradation by measuring the weight loss before and after the reaction, which was calculated as

$$\mathrm{Weight}\;\mathrm{Loss}\;\left(\%\right)=\frac{m_{i}-m_{r}}{m_{i}}\;\times 100,$$

where *m*_i_ is the initial sample weight (g), and *m*_r_ is the weight of HDPE residue (g).

### 4.3 Analytical Methods

The mechanism of PMS activation on Ni@C was investigated by an EPR instrument from Bruker. DMPO was selected as the spin‐trapping agent for SO_4_^•−^ and ·OH during PMS activation. To identify plastic degradation products, we employed a Shimadzu GC‐MS/MS TQ8040 system operating in Electron Ionization (EI) mode, equipped with a RESTEK Rxi‐5Sil MS column (30 m × 0.25 mm, 0.25 µm). Helium was used as the carrier gas, and argon served as the collision‐induced dissociation (CID) gas. The column oven was initially set at 30°C, with an injection temperature of 200°C. Total and column flows were maintained at 5 and 1 mL/min, respectively. The ion source and interface temperatures were set at 230°C and 250°C, respectively. The event time was 0.3 s, and the total run time was 34 min. The temperature program for the oven was as follows: held at 30°C for 0.5 min, ramped to 300°C at 15°C/min, held for 10 min, and then returned to the initial temperature. Detection was conducted in positive ion mode using a Q1 scan. The mass range monitored was *m*/*z* 2–500. All analyses were performed in duplicate. Data processing was performed using Shimadzu LabSolutions software version 4.54. Injection was performed in Splitless mode for the low‐concentration liquid samples, with an injection volume of 5 µL. Blank air and dichloromethane: n‐hexane were injected between groups of samples to reduce carry‐over. The gas sample was analyzed using an online gas chromatograph (Agilent, GC‐8890) equipped with a flame ionization detector (FID) and a thermal conductivity detector (TCD).

### 4.4 Toxicity Assay

For the toxicity tests, the reaction solution was centrifuged and filtered to remove the HDPE and Ni@C catalysts. 10 seeds of mung beans sterilized with 3% NaClO were placed in Petri plates containing a moistened paper towel with 5 mL of DI water and the reaction solution, respectively. The incubation of the respective containers was carried out in the dark at room temperature for 7 days. The plants were then separated from the container, and the lengths of the roots and shoots were measured.

Other experimental details can be found in the Supporting Information.

## Author Contributions

**Xiao Lu** designed and performed experiments and drafted the manuscript. **Shiying Ren**, **Jingkai Lin**, **Kunsheng Hu**, **and Yalan Mo** assisted with the chemical probe, EPR, and HRTEM characterizations. All authors contribute in the paper discussion and revision. **Wenjie Tian and Xiaoguang Duan** supervised the project. The authors would also acknowledge Nobuyuki Kawashima and Ashley Slattery at the Adelaide Microscopy for their support, as well as Brendan Hillary, Ay Ching Hee, and Marzieh Amirmostofian for their technical assistance.

## Conflicts of Interest

The authors declare no conflicts of interest.

## Supporting information




**Supporting File**: adma74578‐sup‐0001‐SuppMat.docx.

## Data Availability

The data that support the findings of this study are available from the corresponding author upon reasonable request.

## References

[1] Plastics—the fast facts 2025. Plastics Europe (2025).

[2] L. Wimberger , G. Ng , and C. Boyer , “Light‐Driven Polymer Recycling to Monomers and Small Molecules,” Nature Communications 15 (2024): 2510, 10.1038/s41467-024-46656-3. PMC10954676 38509090

[3] J. Chen , J. Wu , P. C. Sherrell , et al., “How to Build a Microplastics‐Free Environment: Strategies for Microplastics Degradation and Plastics Recycling,” Advanced Science 9 (2022): 2103764, 10.1002/advs.202103764. PMC8867153 34989178

[4] R. Geyer , J. R. Jambeck , and K. L. Law , “Production, Use, and Fate of All Plastics Ever Made,” Science Advances 3 (2017): 1700782, 10.1126/sciadv.1700782. PMC5517107 28776036

[5] Y. Fu , Z. Guo , F. Pan , Y. Cai , J. Wu , and B. Wang , “Distribution Characteristics and Release Mechanisms of Pb in Surface Sediments in Different Aquatic Environments,” Journal of Contaminant Hydrology 235 (2020): 103704, 10.1016/j.jconhyd.2020.103704. 32896763

[6] J. W. Cottom , E. Cook , and C. A. Velis , “A Local‐to‐Global Emissions Inventory of Macroplastic Pollution,” Nature 633 (2024): 101–108, 10.1038/s41586-024-07758-6. PMC11374682 39232151

[7] W. Tian , P. Song , H. Zhang , et al., “Microplastic Materials in the Environment: Problem and Strategical Solutions,” Progress in Materials Science 132 (2023): 101035, 10.1016/j.pmatsci.2022.101035.

[8] H. Li , J. Wu , Z. Jiang , et al., “Hydroformylation of Pyrolysis Oils to Aldehydes and Alcohols from Polyolefin Waste,” Science 381 (2023): 660–666, 10.1126/science.adh1853. 37561862

[9] J. Zhao , B. Liu , L. Xiong , et al., “Highly Selective Upcycling of Plastic Mixture Waste by Microwave‐Assisted Catalysis over Zn/b‐ZnO,” Nature Communications 16 (2025): 176, 10.1038/s41467-024-55584-1. PMC11836401 39966353

[10] J. Gao , J. Zhao , Z. Xing , et al., “Microwave‐Powered Liquid Metal Degradation of Polyolefins,” Advanced Materials 37 (2025): 2412539, 10.1002/adma.202412539. 39696906

[11] T. Ji , R. Tu , L. Li , et al., “Localizing Microwave Heat by Surface Polarization of Titanate Nanostructures for Enhanced Catalytic Reaction Efficiency,” Applied Catalysis B: Environmental 227 (2018): 266–275, 10.1016/j.apcatb.2017.12.073.

[12] J. Qin , F. Wu , Y. Dou , D. Zhao , C. Hélix‐Nielsen , and W. Zhang , “Advanced Catalysts for the Chemical Recycling of Plastic Waste,” Advanced Materials 37 (2025): 2418138, 10.1002/adma.202418138. 39748624

[13] J. Kang , L. Zhou , X. Duan , H. Sun , Z. Ao , and S. Wang , “Degradation of Cosmetic Microplastics via Functionalized Carbon Nanosprings,” Matter 1 (2019): 745–758, 10.1016/j.matt.2019.06.004.

[14] F. Miao , Y. Liu , M. Gao , et al., “Degradation of Polyvinyl Chloride Microplastics via an Electro‐Fenton‐Like System with a TiO_2_/Graphite Cathode,” Journal of Hazardous Materials 399 (2020): 123023, 10.1016/j.jhazmat.2020.123023. 32535518

[15] K. Hu , P. Zhou , Y. Yang , et al., “Degradation of Microplastics by a Thermal Fenton Reaction,” ACS ES&T Engineering 2 (2022): 110–120, 10.1021/acsestengg.1c00323.

[16] J. Ye , N. Li , W. Gao , et al., “Bimetal‐Carbon Engineering for Polypropylene Conversion into Hydrocarbons and Ketones in a Fenton‐Like System,” Applied Catalysis B: Environment and Energy 358 (2024): 124411, 10.1016/j.apcatb.2024.124411.

[17] J. Lin , K. Hu , Y. Wang , et al., “Tandem Microplastic Degradation and Hydrogen Production by Hierarchical Carbon Nitride‐Supported Single‐Atom Iron Catalysts,” Nature Communications 15 (2024): 8769, 10.1038/s41467-024-53055-1. PMC11464750 39384850

[18] M. Qin , L. Zhang , and H. Wu , “Dielectric Loss Mechanism in Electromagnetic Wave Absorbing Materials,” Advanced Science 9 (2022): 2105553, 10.1002/advs.202105553. PMC8981909 35128836

[19] Z. Wu , H. W. Cheng , C. Jin , et al., “Dimensional Design and Core–shell Engineering of Nanomaterials for Electromagnetic Wave Absorption,” Advanced Materials 34 (2022): 2107538, 10.1002/adma.202107538. 34755916

[20] Q. Li , X. Zhao , L. Xu , et al., “Engineering Strategies in Low‐Dimensional Microwave Absorbers: Fundamentals, Progress, and Outlook,” Materials Science and Engineering: R: Reports 159 (2024): 100795, 10.1016/j.mser.2024.100795.

[21] B. Zhao , Y. Du , Z. Yan , et al., “Structural Defects in Phase‐Regulated High‐Entropy Oxides toward Superior Microwave Absorption Properties,” Advanced Functional Materials 33 (2023): 2209924, 10.1002/adfm.202209924.

[22] J. Yan , Y. Huang , Y. Yan , J. Ding , and P. Liu , “High‐Performance Electromagnetic Wave Absorbers Based on Two Kinds of Nickel‐Based MOF‐Derived Ni@C Microspheres,” ACS Applied Materials & Interfaces 11 (2019): 40781–40792, 10.1021/acsami.9b12850. 31588726

[23] M. Huang , L. Wang , W. You , and R. Che , “Single Zinc Atoms Anchored on MOF‐Derived N‐Doped Carbon Shell Cooperated with Magnetic Core as an Ultrawideband Microwave Absorber,” Small 17 (2021): 2101416, 10.1002/smll.202101416. 34159720

[24] N. Sun , S. S. A. Shah , Z. Lin , Y. Z. Zheng , L. Jiao , and H. L. Jiang , “MOF‐Based Electrocatalysts: An Overview from the Perspective of Structural Design,” Chemical Reviews 125 (2025): 2703–2792, 10.1021/acs.chemrev.4c00664. 40070208

[25] Z. Fan , P. He , H. Bai , et al., “Green Recycling of Waste Poly(ethylene terephthalate) into Ni‐MOF Nanorod for Simultaneous Interfacial Solar Evaporation and Photocatalytic Degradation of Organic Pollutants,” EcoMat 6 (2024): e12422, 10.1002/eom2.12422.

[26] W. P. R. Deleu , I. Stassen , D. Jonckheere , R. Ameloot , and D. E. De Vos , “Waste PET (bottles) as a Resource or Substrate for MOF Synthesis,” Journal of Materials Chemistry A 4 (2016): 9519–9525, 10.1039/C6TA02381A.

[27] J. Li , S. Zhang , Y. Hua , et al., “Facile Synthesis of Accordion‐Like Porous Carbon from Waste PET Bottles‐Based MIL‐53(Al) and Its Application for High‐Performance Zn‐Ion Capacitor,” Green Energy & Environment 9 (2024): 1138–1150, 10.1016/j.gee.2023.01.002.

[28] K. Song , X. Qiu , B. Han , S. Liang , and Z. Lin , “Efficient Upcycling Electroplating Sludge and Waste PET into Ni‐MOF Nanocrystals for the Effective Photoreduction of CO_2_ ,” Environmental Science: Nano 8 (2021): 390–398, 10.1039/D0EN01061H.

[29] C. Yang , H. Wu , J. Yun , et al., “Engineering of Defective MOF‐801 Nanostructures within Macroporous Spheres for Highly Efficient and Stable Water Harvesting,” Advanced Materials 35 (2023): 2210235, 10.1002/adma.202210235. 37219533

[30] N. A. Khan and S. H. Jhung , “Synthesis of Metal‐Organic Frameworks (MOFs) with Microwave or Ultrasound: Rapid Reaction, Phase‐Selectivity, and Size Reduction,” Coordination Chemistry Reviews 285 (2015): 11–23, 10.1016/j.ccr.2014.10.008.

[31] C. Carrillo‐Carrión , J. Farrando‐Perez , L. L. Daemen , Y. Q. Cheng , A. J. Ramirez‐Cuesta , and J. Silvestre‐Albero , “Zr‐Porphyrin Metal–Organic Framework as Nanoreactor for Boosting the Formation of Hydrogen Clathrates,” Angewandte Chemie International Edition 63 (2024): e202315280, 10.1002/anie.202315280. 38088497

[32] M. A. Artsiusheuski , N. P. M. Casati , A. H. Clark , et al., “Controlling the Mechanism of Nucleation and Growth Enables Synthesis of UiO‐66 Metal–Organic Framework with Desired Macroscopic Properties,” Angewandte Chemie International Edition 64 (2025): e202415919, 10.1002/anie.202415919. 39607283

[33] J. He , L. Luo , J. Li , et al., “Exploiting Lattice Carbon‐Induced Modulation Effect in Nickel towards Efficient Alkaline Hydrogen Oxidation,” Angewandte Chemie 137 (2025): e202423647, 10.1002/ange.202423647. 39806266

[34] D. Xu , Y. Pan , M. Chen , et al., “Synthesis and Application of a MOF‐Derived Ni@C Catalyst by the Guidance from an In Situ Hot Stage in TEM,” RSC advances 7 (2017): 26377–26383, 10.1039/C7RA03162A.

[35] A. Dahal and M. Batzill , “Graphene–Nickel Interfaces: A Review,” Nanoscale 6 (2014): 2548–2562, 10.1039/c3nr05279f. 24477601

[36] J. Zhao , B. Chen , and F. Wang , “Shedding Light on the Role of Misfit Strain in Controlling Core–Shell Nanocrystals,” Advanced Materials 32 (2020): 2004142, 10.1002/adma.202004142. 33051904

[37] S. Zhao , Y. Wang , J. Dong , et al., “Ultrathin Metal–Organic Framework Nanosheets for Electrocatalytic Oxygen Evolution,” Nature Energy 1 (2016): 16184, 10.1038/nenergy.2016.184.

[38] Z. Ni and R. I. Masel , “Rapid Production of Metal−Organic Frameworks via Microwave‐Assisted Solvothermal Synthesis,” Journal of the American Chemical Society 128 (2006): 12394–12395, 10.1021/ja0635231. 16984171

[39] M. Wang , X. Dong , Z. Meng , et al., “An Efficient Interfacial Synthesis of Two‐Dimensional Metal–Organic Framework Nanosheets for Electrochemical Hydrogen Peroxide Production,” Angewandte Chemie International Edition 60 (2021): 11190–11195, 10.1002/anie.202100897. 33694245

[40] L. Sun , X. Pan , Y. N. Xie , et al., “Accelerated Dynamic Reconstruction in Metal–Organic Frameworks with Ligand Defects for Selective Electrooxidation of Amines to Azos Coupling with Hydrogen Production,” Angewandte Chemie 136 (2024): e202402176, 10.1002/ange.202402176. 38470010

[41] H. Xu , D. J. Zheng , S. Wang , et al., “Stable Metal–Organic Electrocatalysts for Anion‐Exchange Membrane Water Electrolyzers by Defect Engineering,” Journal of the American Chemical Society 147 (2025): 29838–29851, 10.1021/jacs.5c06156. 40785199

[42] Q. Du , C. Zhu , Z. Yin , et al., “Stacking Effects on Electron–Phonon Coupling in Layered Hybrid Perovskites via Microstrain Manipulation,” ACS Nano 14 (2020): 5806–5817, 10.1021/acsnano.0c00907. 32293867

[43] L. Zhang , S. Li , H. Sun , et al., “Manipulating Lone‐Pair‐Driven Luminescence in 0D Tin Halides by Pressure‐Tuned Stereochemical Activity from Static to Dynamic,” Angewandte Chemie International Edition 62 (2023): e202311912, 10.1002/anie.202311912. 37794619

[44] Y. Miao , Y. Zhao , S. Zhang , R. Shi , and T. Zhang , “Strain Engineering: A Boosting Strategy for Photocatalysis,” Advanced Materials 34 (2022): 2200868, 10.1002/adma.202200868. 35304927

[45] H. Hu , J. Wang , B. Cui , et al., “Atomically Dispersed Selenium Sites on Nitrogen‐Doped Carbon for Efficient Electrocatalytic Oxygen Reduction,” Angewandte Chemie 134 (2022): e202114441, 10.1002/ange.202114441. 34806271

[46] Y. Yan , J. Zhou , C. Li , et al., “Interfacial Coupling Effects in Two‐Dimensional Ordered Arrays for Microwave Attenuation,” Nature Communications 16 (2025): 202, 10.1038/s41467-024-55776-9. PMC11696289 39747194

[47] M. Qiao , X. Lei , Y. Ma , et al., “Application of Yolk–Shell Fe_3_O_4_@N‐Doped Carbon Nanochains as Highly Effective Microwave‐Absorption Material,” Nano Research 11 (2018): 1500–1519, 10.1007/s12274-017-1767-0.

[48] G. Yu , G. Shao , R. Xu , Y. Chen , X. Zhu , and X. Huang , “Metal–Organic Framework‐Manipulated Dielectric Genes Inside Silicon Carbonitride toward Tunable Electromagnetic Wave Absorption,” Small 19 (2023): 2304694, 10.1002/smll.202304694. 37455351

[49] B. Shi , H. Liang , Z. Li , S. Gao , Q. Chang , and H. Wu , “Surface Coordination Engineering of Fe_3_O_4_ with Carboxylic Acids: Tuning Magnetic Permeability for Wideband C‐Band Microwave Absorption,” Carbon 258 (2026): 121832, 10.1016/j.carbon.2026.121832.

[50] Y. Tsukahara , A. Higashi , T. Yamauchi , et al., “In Situ Observation of Nonequilibrium Local Heating as an Origin of Special Effect of Microwave on Chemistry,” The Journal of Physical Chemistry C 114 (2010): 8965–8970, 10.1021/jp100509h.

[51] T. Ano , S. Tsubaki , A. Liu , et al., “Probing the Temperature of Supported Platinum Nanoparticles under Microwave Irradiation by In Situ and Operando XAFS,” Communications Chemistry 3 (2020): 86, 10.1038/s42004-020-0333-y. PMC9814256 36703448

[52] B. Zhao , Y. Du , Z. Yan , et al., “Structural Defects in Phase‐Regulated High‐Entropy Oxides toward Superior Microwave Absorption Properties,” Advanced Functional Materials 33 (2023): 2209924, 10.1002/adfm.202209924.

[53] X. Li , L. G. Tabil , I. N. Oguocha , and S. Panigrahi , “Thermal Diffusivity, Thermal Conductivity, and Specific Heat of Flax Fiber–HDPE Biocomposites at Processing Temperatures,” Composites Science and Technology 68 (2008): 1753–1758, 10.1016/j.compscitech.2008.02.016.

[54] W. Wu , P. Wen , Y. Wang , et al., “Thermal Stimulation Unlocks Ligand‐Dependent Selective Pathways in Cu (II)/PMS Oxidation of Recalcitrant Phosphonates,” Water Research 290 (2026): 125141, 10.1016/j.watres.2025.125141. 41389418

[55] K. Z. Huang and H. Zhang , “Direct Electron‐Transfer‐Based Peroxymonosulfate Activation by Iron‐Doped Manganese Oxide (δ‐MnO_2_) and the Development of Galvanic Oxidation Processes (GOPs),” Environmental Science & Technology 53 (2019): 12610–12620, 10.1021/acs.est.9b03648. 31601099

[56] W. Huang , S. Xiao , H. Zhong , M. Yan , and X. Yang , “Activation of Persulfates by Carbonaceous Materials: A Review,” Chemical Engineering Journal 418 (2021): 129297, 10.1016/j.cej.2021.129297.

[57] S. Zhong , H. Zhou , S. Ren , et al., “Pulse‐Driven Electrocatalysis with Engineered Wooden Electrode for High‐Efficiency, Energy‐Saving and Sustainable Water Treatment,” Nature Water 3 (2025): 890–901, 10.1038/s44221-025-00466-z.

[58] H. Chen , R. Yin , and M. Zhu , “How to Enhance Persulfate Processes by External‐Field Effects: From Fundamentals to Applications,” Water Research 274 (2025): 123026, 10.1016/j.watres.2024.123026. 39740330

[59] S. Garg and T. D. Waite , “Is Singlet Oxygen an Important Oxidant in Advanced Oxidation Processes?,” Environmental Science & Technology 59 (2025): 26320–26341, 10.1021/acs.est.5c09597. PMC12713729 41346183

[60] S. Kanomi , H. Marubayashi , T. Miyata , and H. Jinnai , “Reassessing Chain Tilt in the Lamellar Crystals of Polyethylene,” Nature Communications 14 (2023): 5531, 10.1038/s41467-023-41138-4. PMC10514264 37735491

[61] P. Liu , L. Qian , H. Wang , et al., “New Insights into the Aging Behavior of Microplastics Accelerated by Advanced Oxidation Processes,” Environmental Science & Technology 53 (2019): 3579–3588, 10.1021/acs.est.9b00493. 30829479

[62] E. Syranidou , K. Karkanorachaki , D. Barouta , et al., “Relationship between the Carbonyl Index (CI) and Fragmentation of Polyolefin Plastics during Aging,” Environmental Science & Technology 57 (2023): 8130–8138, 10.1021/acs.est.3c01430. 37194994

[63] J. V. Gulmine , P. R. Janissek , H. M. Heise , and L. Akcelrud , “Polyethylene Characterization by FTIR,” Polymer Testing 21 (2002): 557–563, 10.1016/S0142-9418(01)00124-6.

[64] I. Noda , “Close‐Up View on the Inner Workings of Two‐Dimensional Correlation Spectroscopy,” Vibrational Spectroscopy 60 (2012): 146–153, 10.1016/j.vibspec.2012.01.006.

[65] S. Yue , Z. Zhao , T. Zhang , F. Li , K. Liu , and S. Zhan , “Selective Photoreforming of Waste Plastics into Diesel Olefins via Single Reactive Oxygen Species,” Angewandte Chemie International Edition 63 (2024): e202406795, 10.1002/anie.202406795. 38708785

[66] F. Xu , B. Wang , D. Yang , J. Hao , Y. Qiao , and Y. Tian , “Thermal Degradation of Typical Plastics under High Heating Rate Conditions by TG‐FTIR: Pyrolysis Behaviors and Kinetic Analysis,” Energy Conversion and Management 171 (2018): 1106–1115, 10.1016/j.enconman.2018.06.047.

[67] S. Feng , P. T. Nguyen , X. Ma , and N. Yan , “Photorefinery of Biomass and Plastics to Renewable Chemicals using Heterogeneous Catalysts,” Angewandte Chemie International Edition 63 (2024): e202408504, 10.1002/anie.202408504. 38884612

