# Supplementary material for: Defect‐Engineered Microwave‐Responsive Ni@C Composites From Waste PET for Catalytic Plastic Upcycling
Source: Adv Mater. 2026 Aug 12;38(57):e74578. doi: 10.1002/adma.74578 (PMC13660829; doi:10.1002/adma.74578)
Supplement: Supplementary file 1 [file ADMA-38-e74578-s001.docx]

**Supplemental Information**

Defect-Engineered Microwave-Responsive Ni@C Composites from Waste PET for Catalytic Plastic Upcycling

*Xiao Lu^a^, Shiying Ren^a^, Jingkai Lin^a^, Kunsheng Hu^a^, Yalan Mo^a^, Shaobin Wang^a^, Wenjie Tian^a,^*, Xiaoguang Duan^a,^**

^a^ School of Chemical Engineering, Adelaide University, Adelaide, SA 5005, Australia.

*Corresponding authors:

*E-mail address: wenjie.tian@adelaide.edu.au,* [*xiaoguang.duan@adelaide.edu.au*](mailto:xiaoguang.duan@adelaide.edu.au)

Supplemental Experimental Procedures

Text S1

Materials and chemicals

Waste poly(ethylene terephthalate) (PET) bottles were cut into pieces smaller than 5 mm. High-density polyethylene (HDPE), low-density polyethylene (LDPE), polypropylene (PP), polyvinyl chloride (PVC), polystyrene (PS), acrylonitrile butadiene styrene (ABS) and polycarbonates (PC) were purchased from Sigma Aldrich. Nickle nitrate hexahydrate (Ni(NO_3_)_2_·6H_2_O, >94.5%), N,N-dimethylformamide (DMF, 99.8%), potassium peroxymonosulfate (2KHSO_5_·3KHSO_4_·K_2_SO_4_, Oxone), 5,5-dimethyl-1-pyrroline N-oxide (DMPO, ≥ 97.0%), methanol (MeOH), tert-butyl alcohol (TBA), benzoic acid (BA, ≥ 99.5%), sodium hypochlorite (NaClO), dichloromethane (DCM), n-Hexane, and sulfuric acid are all of an analytical grade and supplied by Sigma. Ultra-pure water (18.2 MΩ cm^−1^) was used in all of the experiments.

Catalyst and samples characterizations

The morphologies of catalysts and microplastics were investigated with field emission scanning electron microscopy (FE-SEM, Zeiss Neon 40 EsB). Integrated differential phase contrast (iDPC) images were measured on the FEI Titan Themis 80–200. High-angle annular dark field (HAADF) and energy dispersive X-ray spectrometry (EDX) images were conducted on JEOL 2100. The copper lacey grid with a diameter of 3.05 mm and square meshes to support the samples for transmission electron microscopy (TEM) and EDX analyses. X-ray diffraction (XRD) analysis was performed on a Rigaku MiniFlex 600 using a Cu-Kα radiation with λ at 1.5418 Å. X-ray photoelectron spectroscopy (XPS) was carried out on a Thermo ESCALAB 250 XPS microscope with monochromatic Al-Kα X-rays at a photon energy of 1486.7 eV. Raman spectra was recorded on a Raman spectrometer (Horiba LabRAM HR Evolution) with a 532 nm green laser. Fourier transform infrared spectrophotometer (FTIR) was conducted on Perkin Elmer Spectrum 400. The specific surface areas of carbon samples were measured using the Brunauer−Emmett−Teller (BET) method from N_2_ adsorption−desorption isotherms (V-Sorb 2800 P, Ultrametrics). Netzsch STA449 F3 Jupiter Simultaneous Thermal Analyzer (STA) coupled with a QMS 403 Aeolos Quadro quadrupole mass spectrometer was used for thermal gravimetric analysis (TGA) and mass spectrometry (MS). The Bruker Magnettech ESR5000 was used to detect free electrons in Ni-MOF and to identify free radicals with DMPO as the spin trapping agent. The X-ray absorption data were collected on the X-ray absorption spectroscopy (XAS) beamline at Australian Synchrotron (Melbourne). All X-ray absorption spectra were measured in a transition mode at room temperature. Ni foil and NiO were regarded as the standard references. All the spectra were collected under ambient conditions, data was analyzed using standard methods. The resulting spectra were energy calibrated, background corrected and normalized at the height of the edge step using the ATHENA module in the IFEFFIT packages.^[1]^ The magnetization behavior of the samples was measured using an Oxford vibrating sample magnetometer (VSM) 3001 at room temperature. The concentration of BA was analyzed using ultrahigh performance liquid chromatography (HPLC, Ultimate 3000, Thermo Scientific) with a C-18 column.

**Figure S1. Experimental setup.** ETHOS EASY microwave reaction system, the sealed reactor vessel, alongside the digital control interface displaying the real-time temperature and microwave power output.

Supplemental Experimental Results

**Table S1. Summary of material and reaction system designations**

| **Designation** | **Category** | **Definition** |
| --- | --- | --- |
| Ni-MOF-HT | Precursor | Ni-MOF synthesized via the hydrothermal route |
| Ni-MOF-MW | Precursor | Ni-MOF synthesized via the microwave-assisted route |
| Ni@C | Catalyst (generic) | Ni-core/carbon-shell composite used for general reference |
| Ni@C-HT | Catalyst series | Ni@C series derived from Ni-MOF-HT |
| Ni@C-MW | Catalyst series | Ni@C series derived from Ni-MOF-MW |
| Ni@C-600/700/800/900-HT | Catalyst | Ni@C-HT pyrolyzed at 600, 700, 800, 900°C (number indicates pyrolysis temperature) |
| Ni@C-600/700/800/900-MW | Catalyst | Ni@C-HT pyrolyzed at 600, 700, 800, 900°C |
| MW | Reaction system | Microwave-assisted catalytic degradation utilizing Ni@C-700-MW+PMS under microwave heating |
| MW-PMS | Reaction system | Noncatalytic control utilizing PMS under microwave heating with no catalyst |
| HT | Reaction system | Conventional degradation utilizing Ni@C-700-MW+PMS under oven heating |

**Figure S2. XRD analysis of Ni-MOF-MW and Ni-MOF-HT.** XRD spectra of Ni-MOF-MW and Ni-MOF-HT from 5° to 60° (a) and 8° to 11° (b).

**Figure S3.** iDPC-STEM images of Ni-MOF-MW viewed along [110] direction, highlighting atomic lattice structure and crystallographic.

**Figure S4. Morphology characterization of Ni-MOF-MW and Ni-MOF-HT.** SEM images of Ni-MOF-HT (a) and Ni-MOF-MW (b, c).

**Figure S5. Surface properties analysis.** BET analysis of nitrogen adsorption-desorption isotherms and comparison of BET surface areas presented in inset bar graph.

**Table S2. Specific area and pore volume.**

|  | Ni-MOF-HT | Ni-MOF-MW |
| --- | --- | --- |
| BET surface area (m^2^/g) | 74.65 | 139.73 |
| Average pore size (nm) | 14.53 | 22.84 |
| Pore volume (cm^3^/g) | 0.29 | 0.87 |
| Micropore volume (cm^3^/g) | 0.03 | 0.06 |
| BJH Median pore width (nm) | 9.99 | 17.20 |

Coupled nitrogen sorption and pore structure analysis (**Figure S5** and **Table S2**) show that Ni-MOF-MW has a BET surface area of 139.7  m^2^/g, average pore size of 22.8 nm, and pore volume of 0.87 cm³/g, all markedly higher than the hydrothermal sample. The steeper nitrogen uptake at high relative pressure and the increased median pore widths further support the presence of enlarged mesopores and a high density of structural defects, confirming that microwave synthesis produces a defect-rich Ni-MOF framework.

Supplementary Note 1. Quantifying linker-to-cluster ratio by TGA

The linker stoichiometry of each Ni-MOF sample was quantified by TGA following the literature.^[2]^ To avoid contributions from residue solvent or incomplete decomposition, the plateau mass at 300 ˚C was used as endpoint for the first weight-loss step. On heating to 600 ˚C, Ni-MOF with formula Ni_2_O_2_(BDC) (MW = 313.38 g/mol) is fully oxidized to two equivalents of NiO, while the organic linker is removed:

$${\mathbf{N}\mathbf{i}}_{\mathbf{2}}\mathbf{O}_{\mathbf{2}}\left( \mathbf{C}_{\mathbf{8}}\mathbf{H}_{\mathbf{4}}\mathbf{O}_{\mathbf{4}} \right) \left( s \right)+{7 \mathbf{O}}_{\mathbf{2}} \left( g \right)=2 \mathbf{N}\mathbf{iO} \left( s \right)+{8\mathbf{CO}}_{\mathbf{2}}\left( g \right)+{2\mathbf{H}}_{\mathbf{2}}\mathbf{O} \left( g \right)$$

The mass at 600 ˚C was normalized to 100% (${\%WL}_{End}$). For an ideal crystal, the expected normalized mass at the 300 ˚C plateau is:

${\%WL}_{Ideal,Plat}=\frac{M_{W}of {Ni}_{2}O_{2}\left( B\mathrm{DC} \right)}{M_{W}of 2 N\mathrm{iO}}= \frac{313.38 g/mol}{2 \times74.69 g/mol}\times100\%= 209.79\%$.

Thus, the theoretical weight loss per BDC linker is:

${\%WL}_{Ideal}= {\%WL}_{Ideal,Plat}-{\%WL}_{End}=209.79\%-100\%=109.79\%$.

The experimental linker-to-cluster ratio (${NL}_{Exp}$) is then

${NL}_{Exp}=\frac{{\%WL}_{Exp,Plat}-100}{109.79}$.

**Table S3. Link-to-cluster ratio of Ni-MOF-HT and Ni-MOF-MW**

| Sample | ${\%WL}_{Exp,Plat}$ | ${NL}_{Exp}$ |
| --- | --- | --- |
| Ni-MOF-HT | $\frac{91.11\%}{45.07\%}*100\%=202.15\%$ | $\frac{202.15\%-100\%}{109.79\%}=0.93$ |
| Ni-MOF-MW | $\frac{91.07\%}{38.30\%}*100\%=237.78\%$ | $\frac{237.78\%-100\%}{109.79\%}=1.25$ |

**Figure S6. TGA analysis in oxygen atmosphere.** TGA under oxygen atmosphere (scan rate of 5 °C/min) of Ni-MOF-MW and Ni-MOF-HT, inserted bar chart: linker to Ni ratio of the different samples.

**Figure S7. TGA-MS analysis in nitrogen atmosphere.** TGA-MS curves of Ni-MOF-MW (a) and Ni-MOF-HT (b). Evolved species were identified based on m/z signals: H_2_O (m/z = 18), CO_2_ (m/z = 44), benzene (m/z = 78).

Ni-MOF-MW exhibits two distinct decomposition peaks at around 430 °C and 550 °C, reflecting heterogeneous ligand environments and stepwise breakdown characteristics of a framework rich in missing-cluster defects. In contrast, the Ni-MOF-HT displays a subtle DTG shoulder around 300 °C and a single, sharp CO_2_ peak at approximately 420 °C, suggesting a uniform, defect-poor structure that undergoes one-step collapse after guest molecules are released.

**Figure S8. Electronic structure analysis of catalysts. a,** Normalized Ni K-edge XANES spectra of Ni foil, NiO, Ni-MOF-MW and Ni-MOF-HT. **b,** k^2^-weighted FT–EXAFS spectra of the Ni-MOFs and the related references at the Ni K–edge. **c and d,** Fitted EXAFS in R-space of Ni-MOFs.

**Table S4. EXAFS fitting results of Ni-MOFs.**

| **Sample** | **Shell** | **Bond length**  **(Å)** | **Coordination Number** | **σ^2^**  **(Å^2^)** | **E0 shift**  **(eV)** | **R-factor** |
| --- | --- | --- | --- | --- | --- | --- |
| **Ni-MOF-MW** | Ni–O | 2.05±0.01 | 5.9±0.9 | 0.010±0.002 | -4.1±1.4 | 0.013 |
| **Ni-MOF-HT** | Ni–O | 2.04±0.02 | 6.3±1.0 | 0.011±0.003 | -5.0±1.8 | 0.011 |

a. The value of the amplitude reduction factor (S_0_^2^) lines 0.79; b. Bond length is the interatomic distance; c. CN is the coordination number; d. σ^2^ is Debye-Waller factor (a measure of thermal and static disorder in absorber scatter distance); e. E0 shift is edge-energy shift (the difference between the zero kinetic energy value of the sample and that of the theoretical model); f. R factor is used to value the goodness of the fitting.

Normalized Ni *K*-edge XANES spectra (**Figure S8a**) show the absorption edge of Ni-MOF-MW at slightly higher energy than that of NiO, indicating an average Ni oxidation state marginally above +2. In the FT-EXAFS spectroscopy (**Figure S8b**) the dominant peak at around 1.55 Å corresponds to the first-shell Ni–O coordination.^[3]^ EXAFS fitting shows a single Ni–O shell at 2.05 ± 0.01 Å with a coordination number of 5.9 ± 0.9, confirming missing-cluster defects that introduce lattice distortion in Ni-MOF-MW.

**Figure S9. Morphology characterization of Ni@C-MW catalysts.**

**Figure S10. TEM images and Ni core size distributions of Ni@C-MW.** The average sizes of the Ni cores are denoted in the top of each panel, demonstrating a continuous size expansion with increasing pyrolysis temperature.

**Figure S11. Lattice spacing variation of Ni@C-700-MW. a** and **b,** Intensity profiles across the Ni (111) plane and C (002) plane shown in **Figure 2e**.

**Figure S12. Temperature-dependent lattice evolution of Ni@C catalysts derived from Ni-MOF-HT. a,** XRD patterns of Ni@C-HT after pyrolysis at 600-900 °C. **b,** Two-dimensional contour plot of the Ni (111) reflection for Ni@C-HT. Reference positions for *fcc* Ni (PDF#06-4262) and graphitic carbon (PDF#75-1621) are indicated.

Supplementary Note 2. Detailed calculation processes of Williamson–Hall (WH) analysis

The microstrain was calculated from refined XRD patterns in **Figure S13** according to the WH plots^[4]^:

$\beta cos\theta=4\varepsilon sin\theta+\frac{k\lambda}{D}$	(S1)

where $\beta$ is the corrected full width at half maximum (FWHM) of the diffraction peak, $\theta$ is the diffraction angle, $\varepsilon$ is the microstrain, $k$ is a dimensionless shape factor, $\lambda$ is the wavelength of the X-ray (Cu K$\alpha$, 1.5406 Å), and the $D$ represents the crystallite size. In **Figure S13e-h**, the plots were drawn with $sin\theta$ along the x-axis and $\beta cos\theta$ along the y-axis. From the linear fit result of the data, the microstrain can be extracted from the slope of line and crystallite size can be calculated from the intercept.

The lattice parameter ($a$) of *fcc* Ni was calculated based on the (111) diffraction peak using Bragg’s law and the standard cubic lattice equation:

$d_{hkl}=\frac{\lambda}{2sin\theta}, a=d_{hkl}\sqrt{h^{2}+k^{2}{+l}^{2}}$	(S2)

where $\lambda$ is the wavelength of the X-ray, $\theta$ is the diffraction angle, and $(hkl)$ denotes the Miller indices of the corresponding lattice plane.

To evaluate the degree of lattice distortion under different pyrolysis temperatures, the relative change in lattice parameter was expressed as:

$\frac{\Delta a}{a}=\frac{a_{Cat.}- a_{0}}{a_{0}}$	(S3)

where $a_{0}$ is the reference lattice parameter of unstrained Ni (3.520 Å). A negative $\Delta a/a$ indicated lattice compression, while a positive value implies lattice expansion.

**Figure S13. XRD Rietveld refinement and WH analysis of Ni@C-MW. a-d.** Rietveld-refined XRD patterns of Ni@C-MW pyrolyzed at 600, 700, 800 and 900^o^C. **e-h.** WH plots corresponding to Ni@C-MW catalysts.

**Figure S14. XRD Rietveld refinement results of Ni@C-MW.** Relative lattice parameter change (Δa/a) (a) and Ni(111) peak FWHM (b) of Ni@C-MW catalysts obtained from XRD and WH analysis.

**Figure S15. Local coordination of Ni@C-MW catalysts. a,** Normalized Ni K-edge XANES spectra comparing Ni@C catalysts with Ni foil. **b,** FT-EXAFS spectra of Ni foil and Ni@C-MW series. **c-f,** R-space fitting of the Ni K-edge EXAFS spectrum.

**Figure S16. Local coordination of Ni@C-700-HT. a,** Normalized Ni K-edge XANES spectra comparing Ni@C-700-HT with Ni foil. **b,** FT-EXAFS spectra of Ni foil and Ni@C-700-HT. **c,** R-space fitting of the Ni K-edge EXAFS spectrum.

**Table S5. EXAFS fitting parameters for Ni@C catalysts**

|  | Shell | Bond length  (Å) | Coordination Number | σ^2^  (Å^2^) | E0 shift  (eV) | R-factor |
| --- | --- | --- | --- | --- | --- | --- |
| Ni@C-600-MW | Ni-Ni | 2.48±0.00 | 11.6±0.6 | 0.006±0.000 | 7.4±0.5 | 0.002 |
| Ni@C-700-MW | Ni-Ni | 2.48±0.00 | 11.6±0.5 | 0.006±0.000 | 7.4±0.5 | 0.002 |
| Ni@C-800-MW | Ni-Ni | 2.48±0.00 | 11.8±0.5 | 0.006±0.000 | 7.4±0.5 | 0.002 |
| Ni@C-900-MW | Ni-Ni | 2.48±0.00 | 12.1±0.6 | 0.006±0.000 | 7.3±0.5 | 0.002 |
| Ni@C-700-HT | Ni-Ni | 2.49±0.00 | 12.0±0.6 | 0.006±0.001 | 7.4±0.7 | 0.003 |

a. The value of the amplitude reduction factor (S_0_^2^) lines 0.79.

**Figure S17. XPS spectra and corresponding fittings for Ni 2p_3/2_ and Ni 2p_1/2_ of all samples.**

**Figure S18. Characterization of carbon disorder.** Raman spectra of Ni@C catalysts derived from Ni-MOF-MW (a) and Ni-MOF-HT (b), with corresponding I_D_/I_G_ ratio.

**Figure S19. Multiple defect descriptors trace an inherited defect population from the Ni-MOF precursors to the resulting Ni@C catalysts.** Normalized defectiveness heatmaps compare structural properties for the precursor series in panel (a) and the derived catalyst series in panel (b).

The continuity between the two stages is captured by the integrated defectiveness heatmaps of the precursors and the catalysts (**Figure S19**). To place the various defect descriptors on a common scale, each defect descriptor was independently normalized to a range from 0 to 1 across the samples in each panel, where 1 signifies the most defective state. To ensure that larger values consistently reflect greater defectiveness, the scaling formulas were adapted based on how each parameter responds to defect density. Descriptors that increase with structural disorder, including SSA, linker-to-cluster ratio, Raman I_D_/I_G_, microstrain from Williamson-Hall (WH) analysis, were scaled as $\frac{X-X_{min}}{X_{max}-X_{min}}$. Conversely, metrics that decrease as defects become prominent were scaled as $\frac{X_{max}-X}{X_{max}-X_{min}}$. These include the Ni–O and Ni–Ni coordination number (CN) and Ni 2p_3/2_ binding energy, where lower values indicate an undercoordinated and electron-rich state. The qualitative EPR g≈2.003 signal was simply encoded as 1 for defective when present and 0 for non-defective when absent. The resulting normalized defectiveness is presented by cell colour, with the corresponding raw values shown within each cell. Normalization was performed separately for Ni-MOF and Ni@C catalysts descriptors within their specific panels. Collectively, these complementary compositional, electronic, structural, and spectroscopic descriptors provide a unified framework for tracking defect evolution. This defect architecture is initially generated by microwave synthesis of the Ni-MOF-MW precursor and is subsequently inherited by the Ni@C-700-MW during controlled pyrolysis.

Supplementary Note 3. Electromagnetic properties characterization

The electromagnetic parameters from 2 to 18 GHz were measured using a vector network analyzer (VNA, E5071C, Agilent, USA). All samples were mixed with paraffin wax at the ratio of 2:8. The mixture was heated to 80 ^o^C on a heating platform, mixed well and then the samples were formed into a coaxial ring (external diameter, 7.0 mm, internal diameter 3.0 mm, height, 2.0 mm). The temperature-dependent dielectric constant was tested by precision LCR Digital Bridge (TH2826, Tonghui Electronic, China).

Reflection loss value can be calculated according to the transmission line theory.^[5,6]^

$\mu_{r}=\mu^{'}-{j\mu}^{''}$	(S4)

$\varepsilon_{r}=\varepsilon^{'}-{j\varepsilon}^{''}$	(S5)

$Z_{in}=Z_{0}\sqrt{\frac{\mu_{r}}{\varepsilon_{r}}}tanh\left[ j\frac{2\pi fd}{c}\sqrt{\varepsilon_{r}\mu_{r}} \right]$	(S6)

$R_{L}=20\log\left| \frac{Z_{in}+Z_{0}}{Z_{in}+Z_{0}} \right|$	(S7)

Where $\mu^{'}$ and $\varepsilon^{'}$ are the real part of permeability and permittivity, respectively, describing the material’s ability to store magnetic and electric energy. $j$ is the imaginary unit ($j^{2}=-1$). $\mu^{''}$ and $\varepsilon^{''}$ are the imaginary part of permeability and permittivity, representing the material’s tendency to dissipate magnetic and electric energy, respectively. $\mu_{r}$ and $\varepsilon_{r}$ stand for the complex permeability and complex permittivity, respectively. $Z_{in}$ is the normalized input impedance of the material. $d$ is the material thickness, $f$ is the microwave frequency, and $c$ is the propagation velocity of electromagnetic waves in vacuum.

Based on the dielectric loss and magnetic loss, the attenuation constant (α) was introduced to evaluate attenuation ability:

$\alpha=\frac{\sqrt{2}\pi f}{c}\times\sqrt{(\mu^{''}\varepsilon^{''}-\mu^{'}\varepsilon^{'})+\sqrt{\left( \mu^{''}\varepsilon^{''}-\mu^{'}\varepsilon^{'} \right)^{2}+{(\mu^{'}\varepsilon^{''}+\mu^{''}\varepsilon^{'})}^{2}}}$	(S8)

**Figure S20. Cole-Cole plots of Ni@C-MW catalysts.**

**Figure S21. Magnetization characterization of Ni@C catalysts. a,** Hysteresis loops of Ni@C-MW catalysts. **b,** Enlarged low-field region highlighting coercivity differences among the four samples. **c,** Comparison of remanent magnetization (*Mr*), saturation magnetization (*Ms*) and coercive field (*Hc*) for the Ni@C-MW series.

**Figure S22. Linear correlations linking defect descriptors, dielectric loss (tan δε, 2.45 GHz) and HDPE weight loss across the Ni@C-MW series.** (a-d) Weight loss versus individual descriptors; (e, g) tan δε versus composite defectiveness and microstrain; (f, h) weight loss versus composite defectiveness and tan δε.

To link these structural defects with catalytic performance, we evaluated their correlations across the Ni@C-MW series (**Figure S22**). Key defect descriptors correlate positively with HDPE weight loss (**Figure S22a-d**). To integrate these factors, we formulated a normalized composite defectiveness index by averaging the normalized descriptors. The index scales linearly with dielectric loss (tan δε at 2.45 GHz), which in turn dictates the degradation efficiency. These relationships confirm that inherited defects enhance dielectric loss, promoting the localized microwave energy dissipation that drives plastic degradation.

**Figure S23. Schematic illustration of three reaction systems.**

**Figure S24. Optimization of operational parameters and kinetic evaluation for HDPE degradation.** (a) Time-dependent weight loss of HDPE across the MW-PMS, MW, and HT systems. (b) Corresponding pseudo-first-order kinetic fitting of the degradation profiles. (c–e) Effects of (c) PMS dosage and reaction temperature, (d) initial solution pH, and (e) Ni@C-700-MW catalyst dosage on the degradation efficiency.

**Figure S25. Structural characterization and degradation activity of non-defective control microwave absorbers.** (a, b) XRD patterns of graphite and Fe_3_O_4_. (c) Raman spectra of graphite. (d) HDPE weight loss under identical reaction conditions for Ni@C-MW, graphite and Fe_3_O_4_. All reactions used [catalysts] = 0.1 g/L, [PMS] = 4 g/L, [HDPE] = 1.0 g/L, temperature = 180 °C, pH 2.4, microwave power = 250 W in MW system unless otherwise specified.

XRD confirms the high phase purity of graphite and Fe_3_O_4_, while Raman spectroscopy indicates the low defect density of graphite. ^[7]^ Despite their intrinsic microwave responsiveness, graphite and Fe_3_O_4_ exhibited substantially lower HDPE degradation efficiencies than Ni@C-700-MW.

**Figure S26. Heating rate profile of MW and HT systems.**

**Figure S27. Effect of scavenger dosage on HDPE degradation.**

**Figure S28. Radical identification and open-circuit potential measurements. a,** DMPO spin-trapping EPR spectra for •OH and SO_4_^•−^ at 80 °C. **b**, EPR spectra probing for ^1^O_2_ and ^•^O_2_^−^ at 80 °C. **c,** Quenching experiments using FFA and p-BQ. **d,** Open-circuit potential evolution over time before and after the addition of PMS for the evaluated catalyst series.

**Figure S29. PMS consumption in each reaction system.** Reaction conditions: [Ni@C-700-MW] = 0.1 g/L, [PMS] = 4 g/L, temperature = 180 °C, pH 2.4, microwave power = 250 W in MW system unless otherwise specified.

**Figure S30. Surface morphology of HDPE after 4 h degradation in microwave catalytic system. a,** Overview SEM image. **b,** High-magnification view of the boxed region in a.

**Figure S31. Time-resolved XRD patterns of HDPE.** XRD patterns of HDPE collected at different reaction intervals within the MW systems. The characteristic diffraction peaks of HDPE are observed at 21.6° and 24.0°, corresponding to the (110) and (200) planes, respectively.

**Figure S32. FT-IR spectra and carbonyl evolution during HDPE degradation in the MW system.** Time depended FT-IR spectra at 3500-500 cm^-1^ (a) and 1800-800 cm^-1^ (b). **c,** Carbonyl index calculated as *I*_1715_/*I*_2915_.

Supplementary Note 4. Noda’s rule in two-dimensional correlation spectroscopy (2D-COS) analysis

The signs of cross peaks in both the synchronous and asynchronous maps provide detailed insight into the sequential order of spectral intensity changes induced by external perturbation. Specifically, they indicate which of the two spectral variables, ν₁ or ν₂, responds first. A set of established guidelines, commonly known as Noda’s rules,[8] are widely used to determine this sequence:

(1) If Φ(ν₁, ν₂) and Ψ(ν₁, ν₂) share the same sign (both positive or both negative), the change at ν₁ occurs before the change at ν₂ during the perturbation.

(2) If Φ(ν₁, ν₂) and Ψ(ν₁, ν₂) have opposite signs (one positive and one negative), the spectral intensity at ν₂ changes before that at ν₁.

**Table S6. Signs of cross peaks in synchronous (Φ, outside brackets) and asynchronous spectra (Ψ, inside brackets) for the MW system.**

| Band assignments | Wavenumber (cm^-1^) | Sign* | | | |
| --- | --- | --- | --- | --- | --- |
|  | ν_1_ ν_2_ | 1169 | 1715 | 2847 | 2915 |
| C−O stretching | 1169 | + | +(+) | -(+) | -(+) |
| C=O stretching | 1715 |  | + | -(+) | -(+) |
| –CH_2_– symmetric stretching | 2847 |  |  | + | +(-) |
| –CH_2_– asymmetric stretching | 2915 |  |  |  | + |

Note: (i) “+” means a positive sign; “−” means negative sign. (ii) “*” denotes that signs were obtained in the bottom-right corner of the 2D maps.

**Figure S33. FT-IR spectra and carbonyl evolution during HDPE degradation in MW-PMS system.** Time-dependent FT-IR spectra at 3500-500 cm^-1^ (a) and 1800-800 cm^-1^ (b). **c** and **d,** 2D-COS synchronous and asynchronous FT-IR spectra for HDPE during 0-16 h reaction.

**Table S7. Signs of cross peaks in synchronous (Φ, outside brackets) and asynchronous spectra (Ψ, inside brackets) for MW-PMS system.**

| Band assignments | Wavenumber (cm^-1^) | Sign* | | | |
| --- | --- | --- | --- | --- | --- |
|  | ν_1_ ν_2_ | 1169 | 1715 | 2847 | 2915 |
| C−O stretching | 1169 | + | +(-) | -(-) | -(-) |
| C=O stretching | 1715 |  | + | -(-) | -(-) |
| –CH_2_– symmetric stretching | 2847 |  |  | + | +(+) |
| –CH_2_– asymmetric stretching | 2915 |  |  |  | + |

**Figure S34. FT-IR spectra and carbonyl evolution during HDPE degradation in HT system.** Time-dependent FT-IR spectra at 3500-500 cm^-1^ (a) and 1800-800 cm^-1^ (b). **c** and **d,** 2D-COS synchronous and asynchronous FT-IR spectra for HDPE during 0-16 h reaction.

**Table S8. Signs of cross peaks in synchronous (Φ, outside brackets) and asynchronous spectra (Ψ, inside brackets) for HT system.**

| Band assignments | Wavenumber (cm^-1^) | Sign* | | | |
| --- | --- | --- | --- | --- | --- |
|  | ν_1_ ν_2_ | 1169 | 1715 | 2847 | 2915 |
| C−O stretching | 1169 | + | +(+) | -(-) | -(-) |
| C=O stretching | 1715 |  | + | -(-) | -(-) |
| –CH_2_– symmetric stretching | 2847 |  |  | + | +(+) |
| –CH_2_– asymmetric stretching | 2915 |  |  |  | + |

Supplementary Note 5. Carbon distribution calculations

To accurately quantify the transformation of HDPE into various product phases, all mass values are reported as milligrams of carbon (mg C).^[9]^ HDPE consists of repeating ethylene units ${(C_{2}H_{4})}_{n}$, and the theoretical carbon mass fraction ($\omega_{C}$) is calculated based on the molar masses of Carbon (12.01 g/mol) and Hydrogen (1.01 g/mol):

$\omega_{C}=\frac{2\times12.01}{\left( 2\times12.01 \right)+(4\times1.01)}=\frac{24.02}{28.06}=0.856$.

Thus, the initial carbon loading ($C_{initial})$ is determined by:

$C_{initial}=m_{HDPE}\times0.856$.

For the experimental systems used in this study:

For 1.0 g/L HDPE in 50 mL system: $50 mg HDPE\times0.856=42.8 mg C$.

For 0.5 g/L HDPE in 50 mL system: $25 mg HDPE\times0.856=21.4 mg C$.

The distribution of carbon across the three phases is analyzed using a combination of gravimetric measurements and TOC quantification.

The carbon content of solid phase ($C_{solid}$) is calculated by the mass of unreacted solid residues:

$C_{solid}=m_{HDPE}\times0.856$.

The total carbon content in the aqueous ($C_{liquid})$ is quantified via TOC analysis:

$C_{liquid}={TOC}_{concentration}\times V_{liquid}$.

The gas content in the gas phase is determined by the difference between the initial loading and the sum of the recovered carbon in the solid and liquid phases:

$C_{gas}=C_{initial}-(C_{solid}+C_{liquid})$.

**Figure S35. Time-dependent carbon mass balances.** Evolution of carbon distribution among gas, liquid, and solid phases over time for the MW-0.5 system (a), MW-PMS system (b) and HT system (c).

**Figure S36. GC-MS chromatograms of liquid products from HDPE degradation. a,** Evolution of liquid product distribution over different reaction times in the MW System. **b,** Comparison of GC-MS chromatograms for liquid products obtained from the MW-0.5, MW, HT, and MW-PMS systems.

**Figure S37. Carbon number distribution of liquid products.** Distribution of hydrocarbons and oxygenates as a function of carbon number for the MW-8h (a), MW-16h (b), MW-PMS (c), and MW-0.5 (d) systems.

**Figure S38. Liquid product composition obtained based on chemical functionality. a,** Time-dependent evolution of the chemical categories in the liquid products obtained from the MW system. **b,** Comparison of liquid product composites across different reaction systems.

**Figure S39. Distribution of C_1_-C_4_ light hydrocarbon gaseous products in the MW and MW-0.5 systems.**

**Table S9. Specific liquid products in MW system identified by GC-MS**

| **No.** | **Ret. Time (min)** | **Sample Identification** | **Molecular Formula** | **MW (g/mol)** |
| --- | --- | --- | --- | --- |
| 1 | 3.27 | Heptane, 2-methyl- | C_8_H_18_ | 114 |
| 2 | 3.43 | 3-Hexanone | C_6_H_12_O | 100 |
| 3 | 3.47 | 2-Hexanone | C_6_H_12_O | 100 |
| 4 | 3.55 | 3-Hexanol | C_6_H_14_O | 102 |
| 5 | 3.59 | 2-Pentanol, 4-methyl- | C_6_H_14_O | 102 |
| 6 | 3.79 | Octane, 2-methyl- | C_9_H_20_ | 128 |
| 7 | 4.20 | Octane, 4-methyl- | C_9_H_20_ | 128 |
| 8 | 4.86 | 2-Pentenoic acid, 4-hydroxy- | C_5_H_8_O_3_ | 116 |
| 9 | 4.91 | Octane, 2,7-dimethyl- | C_10_H_22_ | 142 |
| 10 | 4.96 | 4,5-Dipropenyldihydro-furan-2-one | C_10_H_14_O_2_ | 166 |
| 11 | 5.31 | Nonane, 2-methyl- | C_10_H_22_ | 142 |
| 12 | 5.42 | Nonane, 2,5-dimethyl- | C_11_H_24_ | 156 |
| 13 | 5.71 | Undecane | C_11_H_24_ | 156 |
| 14 | 5.78 | Heptane, 2,5,5-trimethyl- | C_10_H_22_ | 142 |
| 15 | 5.85 | Hexane, 2,4-dimethyl- | C_8_H_18_ | 114 |
| 16 | 5.96 | Nonane, 2,3-dimethyl- | C_11_H_24_ | 156 |
| 17 | 6.34 | 1-Hexanol, 5-methyl-2-(1-methylethyl)- | C_10_H_22_O | 158 |
| 18 | 6.80 | Nonane, 4,5-dimethyl- | C_11_H_24_ | 156 |
| 19 | 7.48 | Undecane, 2-methyl- | C_12_H_26_ | 170 |
| 20 | 7.87 | Dodecane, 2-methyl- | C_13_H_28_ | 184 |
| 21 | 7.99 | Allyl n-octyl ether | C_11_H_22_O | 170 |
| 22 | 8.07 | Dodecane, 4-methyl- | C_13_H_28_ | 184 |
| 23 | 8.16 | Decane, 2,8,8-trimethyl- | C_13_H_28_ | 184 |
| 24 | 8.26 | Dodecane, 2,6,11-trimethyl- | C_15_H_32_ | 212 |
| 25 | 8.39 | Dodecane, 2,6,10-trimethyl- | C_15_H_32_ | 212 |
| 26 | 8.48 | Tetradecane, 5-methyl- | C_15_H_32_ | 212 |
| 27 | 8.63 | 4-Undecanone | C_11_H_22_O | 170 |
| 28 | 8.69 | Dodecane, 4,6-dimethyl- | C_14_H_30_ | 198 |
| 29 | 8.76 | Decane, 3,3,8-trimethyl- | C_13_H_28_ | 184 |
| 30 | 8.81 | Tridecane, 4-methyl- | C_14_H_30_ | 198 |
| 31 | 8.90 | Decane, 2,3,7-trimethyl- | C_13_H_28_ | 184 |
| 32 | 9.20 | 3-Ethyl-2,6,10-trimethylundecane | C_16_H_34_ | 226 |
| 33 | 9.29 | Pentadecane | C_15_H_32_ | 212 |
| 34 | 9.43 | Hexadecane | C_16_H_34_ | 226 |
| 35 | 9.50 | Heptadecane | C_17_H_36_ | 240 |
| 36 | 9.77 | 9-methylheptadecane | C_18_H_38_ | 254 |
| 37 | 9.91 | Tetradecane, 4-methyl- | C_15_H_32_ | 212 |
| 38 | 10.38 | Tetradecane, 2,6,10-trimethyl- | C_17_H_36_ | 240 |
| 39 | 10.58 | Dodecane, 1-methoxy- | C_13_H_28_O | 200 |
| 40 | 10.92 | Hexadecane, 2,6,11,15-tetramethyl- | C_20_H_42_ | 282 |
| 41 | 11.10 | Pentadecane, 4-methyl- | C_16_H_34_ | 226 |
| 42 | 11.25 | Hexadecane, 7,9-dimethyl- | C_18_H_38_ | 254 |
| 43 | 11.39 | γ-tetradecalactone | C_14_H_26_O2 | 226 |
| 44 | 11.67 | 5,5-Diethylpentadecane | C_19_H_40_ | 268 |
| 45 | 11.83 | Hexadecane, 2,6,10,14-tetramethyl- | C_20_H_42_ | 282 |
| 46 | 12.27 | 5,8-Tridecadione | C_13_H_24_O_2_ | 212 |
| 47 | 12.45 | Heptadecane, 8-methyl- | C_18_H_38_ | 254 |
| 48 | 12.70 | Heptadecane, 2-methyl- | C_16_H_34_ | 226 |
| 49 | 12.84 | 9-Heptadecanone | C_17_H_34_O | 254 |
| 50 | 12.99 | Heneicosane | C_21_H_44_ | 296 |
| 51 | 13.23 | Eicosane | C_20_H_42_ | 282 |
| 52 | 13.64 | Tetracosane | C_24_H_50_ | 338 |
| 53 | 13.71 | 2-Methylpentacosane | C_26_H_54_ | 366 |
| 54 | 13.78 | Carbonic acid, decyl tetradecyl ester | C_25_H_50_O_3_ | 398 |
| 55 | 14.35 | Octacosane | C_28_H_58_ | 394 |
| 56 | 14.65 | Octacosane, 2-methyl- | C_29_H_60_ | 408 |
| 57 | 14.90 | Nonacosane | C_29_H_60_ | 408 |
| 58 | 14.96 | 1,3-Propanediol, docosyl ethyl ether | C_27_H_56_O_2_ | 412 |
| 59 | 15.14 | Carbonic acid, decyl pentadecyl ester | C_26_H_52_O_3_ | 412 |
| 60 | 15.39 | Isopropyl hexacosyl ether | C_29_H_60_O | 424 |
| 61 | 15.52 | Nonacosanal | C_29_H_58_O | 422 |
| 62 | 15.59 | Triacontane | C_30_H_62_ | 422 |
| 63 | 15.93 | Nonacosane, 2-methyl- | C_30_H_62_ | 422 |
| 64 | 16.39 | Docosyl nonyl ether | C_31_H_64_O | 452 |
| 65 | 17.09 | 2-Methylhexacosane | C_27_H_56_ | 380 |
| 66 | 17.30 | 2-Methyltriacontane | C_31_H_64_ | 436 |
| 67 | 17.43 | Docosanedioic acid, dimethyl ester | C_24_H_46_O_4_ | 398 |
| 68 | 18.54 | 13-Methylheptacosane | C_28_H_58_ | 394 |
| 69 | 19.67 | Dotriacontane | C_32_H_66_ | 450 |
| 70 | 19.95 | 2-Tritriacontanone | C_33_H_66_O | 478 |

**Table S10. Lab-scale evaluation of energy accounting for each reaction systems**

|  | Energy per minute (kJ/min) | HDPE per batch (g) | Degradation efficiency per batch (%) |
| --- | --- | --- | --- |
| MW-PMS | 15.3 | 0.3 | 32.4 |
| HT | 25.8 | 0.1 | 48.5 |
| MW | 15.3 | 0.3 | 62.9 |
| MW-0.5 | 15.3 | 0.15 | 96.0 |

Note a. Energy per minute is the average power, calculated as the energy per batch divided by the total reaction time (720 min, 12 h). Energy per batch was calculated as (heating power x heating time) + (hold power x hold time). The powers and times were 1000 W for 3.22 min then 250 W for 720 min for MW and MW-0.5, 1000 W for 3.63 min then 250 W for 720 min for MW-PMS, and 1300 W for 13.5 min then 406 W for 720 min for HT.

**Table S11. Products revenue assumptions**

|  |  | MW-PMS | | HT | | MW | | MW-0.5 | |
| --- | --- | --- | --- | --- | --- | --- | --- | --- | --- |
|  | Price ($/t) | Yield (kg/t) | Revenue ($/t) | Yield (kg/t) | Revenue ($/t) | Yield (kg/t) | Revenue ($/t) | Yield (kg/t) | Revenue ($/t) |
| Gasoline | 1130 | 2.3 | 2.5 | 4.9 | 5.6 | 9.6 | 10.8 | 0.8 | 0.9 |
| Diesel | 690 | 6.9 | 4.7 | 19.7 | 13.6 | 28.2 | 19.4 | 11.7 | 8.1 |
| Lube oil | 725 | 22.1 | 16.0 | 69.5 | 50.4 | 22.9 | 16.6 | 81.8 | 59.3 |
| Wax | 1100 | 11.6 | 12.7 | 6.4 | 7.0 | 39.3 | 43.3 | 47.6 | 52.3 |
| H_2_ | 2500 | 1.2 | 3.0 | 0.1 | 0.2 | 9.4 | 23.5 | 19.8 | 49.5 |
| CO | 500 | 145.7 | 72.8 | 144.6 | 72.3 | 294.0 | 147.0 | 514.8 | 257.4 |
| Total revenue |  | 111.9 | | 149.1 | | 260.6 | | 427.5 | |

a. Gasoline: Pricing information referenced from IMARC group naphtha pricing report 2026 edition (https://www.imarcgroup.com/naphtha-pricing-report). Report ID: SR112026A22707

b. Diesel fuel: Pricing information referenced from E-commerce of chemical group report dated November 2025. (https://www.echemi.com/pip/diesel-oil-pd2202071022.html).

c. Lube oil: Pricing information referenced from IMARC group base oil pricing report dated Q3 2025 (https://www.imarcgroup.com/base-oil-pricing-report). Report ID: SR112026A22299

d. Wax: Pricing information referenced from IMARC group paraffin wax prices data report 2026 edition (https://www.imarcgroup.com/paraffin-wax-pricing-report). Report ID: SR112026A22395

e, H_2_: Pricing information referenced from IMARC group hydrogen pricing report 2026 edition (https://www.imarcgroup.com/hydrogen-pricing-report). Report ID: SR112026A22312

f. CO: Pricing information referenced from IMARC Group carbon monoxide prices data report 2026 edition (https://www.imarcgroup.com/carbon-monoxide-price-trend). Report ID: SR112026A38141

**Figure S40. Estimated the Log10 values of acute and chronic toxicity for fish, daphnid and green algae of C_50_H_102_ as model molecular of HDPE and transformation products by ECOSAR.** (Organic chemicals label number as shown in **Table S9**).

Using C_50_H_102_ as a model compound of HDPE, the ECOSAR results are predicted to pose no greater ecological risk than the parent microplastics.

**Figure S41. Toxicity evaluation.** The germination rate and growth status of bean sprouts cultivated with ultrapure water (a1-a5) and plastic degraded solution (b1-b5).

**Table S12. The germination rate and growth status of bean sprouts cultivated with ultrapure water and plastic degraded solution.**

|  | **Control (D.W)** | **MW-0.5** | **MW** |
| --- | --- | --- | --- |
| **Germination** | 100 % | 100 % | 100 % |
| **Shoot (cm)** | 3.1 ± 0.9 | 9.3 ± 1.1 | 9.0 ± 0.6 |
| **Root (cm)** | 8.7 ± 1.7 | 9.7 ± 1.0 | 9.8 ± 1.1 |
| **Germination index** | 100 % | 111% | 113 % |

**Figure S42. Stability tests of Ni@C-700-MW.** Each run was performed under 180 °C for 12h.

**Figure S43. XRD characterization of Ni@C-700-MW after 1-6 runs.**

**Figure S44. Electronic structure analysis of used catalysts.** (a) Normalized Ni *K*-edge XANES spectra of Ni foil, Ni@C-700-MW after 2 and 6 runs. (b) FT-EXAFS spectra of Ni foil and Ni@C-700-MW after 2 and 6 runs. (c) and (d) R-space fitting of the Ni *K*-edge EXAFS spectrum.

**Table S13. EXAFS fitting results of Ni@C-700-MW catalysts after 2 and 6 runs**

| **Sample** | **Shell** | **Bond length**  **(Å)** | **Coordination Number** | **σ^2^**  **(Å^2^)** | **E0 shift**  **(eV)** | **R-factor** |
| --- | --- | --- | --- | --- | --- | --- |
| **Run 2** | Ni-Ni | 2.48±0.00 | 11.3±0.8 | 0.006±0.001 | 7.7±0.7 | 0.014 |
| **Run 6** | Ni-Ni | 2.49±0.01 | 9.6±1.2 | 0.006±0.001 | 7.8±1.3 | 0.014 |

a. The value of the amplitude reduction factor (S_0_^2^) lines 0.79.

**Figure S45. Catalytic degradation efficiency across different plastic feedstocks and concentrations.** (a) Weight loss of HDPE at 0.1 g/L within 1 h. (b) Weight loss of a complex plastic mixture (HDPE, PP, PVC, PET, and PS) at concentration of 0.5 and 1.0 g/L. Reaction conditions: [Ni@C-700-MW] = 0.1 g/L, [PMS] = 4 g/L, temperature = 180 °C, pH 2.4, microwave power = 250 W in MW system unless otherwise specified.

**Table S14. Fenton/Fenton-like system for plastic degradation**

| **Plastic type** | **Method** | **Catalyst and dosage** | **Temperature and reaction time** | **Degradation efficiency** | **Stability** | **Ref.** |
| --- | --- | --- | --- | --- | --- | --- |
| UHMWPE | Hydrothermal Fenton  (H_2_O_2_) | 4 mM FeSO_4_·7H_2_O (homogeneous) | 160°C, 16h | 95.9 wt% weight loss, 75.6% TOC mineralization at 12 h | N.M. | [9] |
| PVC | Hydrothermal Fenton (H_2_O_2_) under subcritical water coupled with flash Joule heating | 29 mM Fe(OH)_3_ (homogeneous) | 250°C, 3h | >99% dehydrochlorination efficiency, | N.M. | [10] |
| PP | Hydrothermal Fenton reaction (PMS) | 0.33 g/L CuMgCWS600 | 160°C, 14h | 77.70 wt% weight loss | 42 h (9.1% reactivity decline) | [11] |
| UHMWPE | Tandem hydrothermal Fenton (H_2_O_2_)/photocatalytic reaction | 1.0 g/L FeSAhCN | 160°C, 12h | 98 wt% weight loss | 72h with no reactivity decline | [12] |
| LDPE/PP | Microwave-activated Fenton reaction (H_2_O_2_) | 1.0 g/L graphite | 200°C, 3h | LDPE: 34 wt% weight loss PP: 14 wt% weight loss | N.M. | [13] |
| PE | Hydrothermal Fenton reaction (PMS) | 1.0 g/L Mn@NCNTs | 120°C, 8h | 44 wt% weight loss | 40h (50% reactivity decline) | [14] |
| HDPE | Microwave-assisted Fenton reaction (PMS) | 0.1 g/L Ni@C-700-MW | 180°C, 12h | 62.9 wt% weight loss for 1.0 g/L; 96 wt% weight loss for 0.5 g/L | 72 h with 12.9% reactivity decline | This work |

UHMWPE: Ultra-High Molecular Weight Polyethylene

PVC: Polyvinyl Chloride

PP: Polypropylene

LDPE: Low-Density Polyethylene

PE: Polyethylene

PMS: Peroxymonosulfate.

N.M.: Not Mentioned

**Figure S46. Reaction route.** The proposed plastic degradation route in Fenton-like reaction in microwave catalytic system.

**Figure S47. Proposed mechanism for HDPE in the MW-PMS and HT systems.**

**Reference**

[1] Ravel, B. & Newville, M. ATHENA , ARTEMIS , HEPHAESTUS : data analysis for X-ray absorption spectroscopy using IFEFFIT. *J. Synchrotron Radiat.* **12**, 537–541 (2005).

[2] Ahmad, B. I. Z. *et al.* Defect-engineered metal-organic frameworks as bioinspired heterogeneous catalysts for amide bond formation. *J. Am. Chem. Soc.* **146**, 34743–34752 (2024).

[3] Liu, Y. *et al.* Molecular engineering of metal-organic frameworks as efficient electrochemical catalysts for water oxidation. *Adv. Mater.* **35**, 2300945 (2023).

[4] Song, J., *et al.* Supramolecular interactions of flexible 2D perovskite in microstrain releasing and optoelectronic properties recovery. *Adv. Funct. Mater.* **32**, 2203329 (2022).

[5] Yan, J., *et al.* High-performance electromagnetic wave absorbers based on two kinds of nickel-based MOF-derived Ni@C microspheres. *ACS Appl. Mater. Interfaces* **11**, 40781-40792 (2019).

[6] Yan, Y., *et al.* Interfacial coupling effects in two-dimensional ordered arrays for microwave attenuation. *Nat. Commun*. **16**, 202 (2025)

[7] Glatthaar, C., Badaczewski, F., Klar, P. J., & Smarsly, B. M. Between order and confusion: Clearing up structural misconceptions in carbon materials nomenclature. *Angew. Chem. Int. Ed.* **65**, e19975 (2026).

[8] Noda, I. Vibrational Spectroscopy Close-up view on the inner workings of two-dimensional correlation spectroscopy. *Vib. Spectrosc.* **60**, 146*-*153 (2012).

[9] Hu, K. *et al.* Degradation of microplastics by a thermal Fenton reaction. *ACS ES&T Engg.* **2**, 110–120 (2022).

[10] Yang, J. *et al.* Hydrothermal Fenton-like process for dehydrochlorination and recovering of PVC pipe microplastics in aquatic systems. *Water Res.* **293**, 125483 (2026).

[11] Ye, J. *et al.* Bimetal-carbon engineering for polypropylene conversion into hydrocarbons and ketones in a Fenton-like system. *Appl. Catal. B Environ.* **358**, 124411 (2024).

[12] Lin, J. *et al.* Tandem microplastic degradation and hydrogen production by hierarchical carbon nitride-supported single-atom iron catalysts. *Nat. Commun.* **15**, 8769 (2024).

[13] Sanz-Abengozar, I. *et al.* Rapid activation of microplastics by microwave heating in an aqueous phase: A novel approach for enhanced plastic recycling. *Sep. Purif. Technol.* **361**, 131347 (2025).

[14] Kang, J. *et al.* Degradation of cosmetic microplastics via functionalized carbon nanosprings. *Matter* **1**, 745-758 (2019).
